# The Role of Chirality-Induced Spin Selectivity in Helicene-Based Photogenerated Radical Pairs

**DOI:** 10.1021/jacs.6c02634

**Published:** 2026-06-13

**Authors:** Giulia Agnoloni, Alessandro Chiesa, Ryan M. Young, Federico Totti, Stefano Menichetti, Caterina Viglianisi, Michael R. Wasielewski, Stefano Carretta, Alberto Privitera, Roberta Sessoli

**Affiliations:** † Department of Chemistry “U. Schiff”, 9300 University of Florence and UdR INSTM Firenze, 50019 Sesto Fiorentino, Italy; ‡ Department of Chemistry, Center for Molecular Quantum Transduction, and Institute for Quantum Information Research and Engineering, 3270 Northwestern University, Evanston, Illinois 60208-3113, United States; § Department of Mathematical, Physical and Computer Sciences, 9370 University of Parma and UdR INSTM Parma, 43124 Parma, Italy; ∥ INFN-Sezione di Milano Bicocca, Gruppo Collegato di Parma, 43124 Parma, Italy; ⊥ Department of Industrial Engineering, University of Florence and UdR INSTM Firenze, 50139 Firenze, Italy

## Abstract

Chirality-induced spin selectivity (CISS) revealed a close connection between molecular chirality and electron spin. Because CISS is observed even at room temperature, it offers a promising route toward spin-based technologies operable under ambient conditions. However, its microscopic origin remains the subject of debate. In recent theories, the parameters governing the electron motion through the chiral bridge play a key role in CISS efficiency. To disentangle this specific contribution from that arising from the overall intrinsic chirality of the molecule, we synthesized a new chiral donor–acceptor dyad (Dχ–B–A) incorporating a thia-bridged[4]­helicene donor, known to have high CISS efficiency in transport experiments, and a perylene diimide (PDI) acceptor connected by a three-ethynylbenzene bridge. Transient absorption measurements at 85 K show that photoexcitation of PDI generates a long-lived radical pair (Dχ^·+^–B–A^·–^) with a lifetime exceeding 500 ns. The combined analysis of the spin polarization mechanism using time-resolved electron paramagnetic resonance, DFT calculations, and theoretical modeling indicates weak CISS polarization and suggests that CISS efficiency is higher in the presence of a chiral bridge.

## Graphical abstract

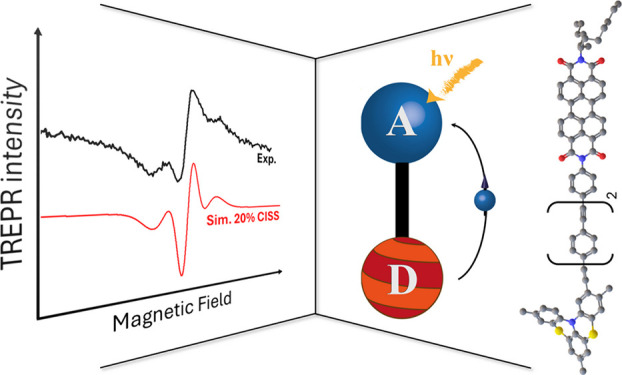

## Introduction

Molecules provide a unique platform for quantum technologies, offering tunable magnetic and electronic properties through chemical design.^1–3^ In this context, chirality provides a powerful means to influence the electron spin degree of freedom. The Chirality-Induced Spin Selectivity (CISS) effect describes the preferential transmission of electrons by chiral molecules based on their spin orientation.^4–7^ The first evidence of CISS was reported in 1999 by Naaman and co-workers, who observed asymmetric scattering of electrons in thin, organized films of chiral molecules on a gold surface.^4^ Since then, numerous experimental and theoretical studies have been conducted to investigate this phenomenon.^5–7^ For applications in quantum technologies based on organic molecules, this phenomenon must manifest at the molecular level, for instance, through an intramolecular photoinduced electron-transfer process within a chiral system.^8,9^ This behavior has been proposed by some of us and later demonstrated by directly probing the spin dynamics of photoinduced electron transfer at the molecular scale.^10–15^ Chirality influences the spin dynamics of spin-correlated radical pairs (SCRPs) produced in donor-chiral bridge-acceptor (D–Bχ–A) molecules through photoinduced electron transfer processes. Using time-resolved electron paramagnetic resonance (TREPR) spectroscopy, Eckvahl et al. demonstrated CISS in systems in which photoexcitation of the donor unit induced electron transfer across the chiral bridge. In the first system studied, the donor was a *peri*-xanthenoxanthene (PXX) unit linked to the naphthalene-1,8:4,5-bis­(dicarboximide) (NDI) acceptor via an axially chiral bridge composed of naphthalene-1,8-dicarboximides.^10^ Selective photoexcitation of the donor is followed by two-step electron transfer. Orientation-dependent TREPR experiments in liquid crystals revealed the formation of the spin-correlated radical pair (SCRP) with distinct spin-polarization features in the chiral dyads compared to achiral analogues. Spectral simulations indicate a CISS efficiency of approximately 50%, consistent with experiments on similar chiral bridges studied using magnetoconductive (mc)-AFM.^16^ CISS was also observed in the hole transfer process through TREPR in D–Bχ–A molecules based on 2,2-dimethoxyoctahydro-1,1’-binaphthalene chiral bridge.^11^ Notably, the observation of CISS was achieved in a butyronitrile glass at 85 K, where the molecules are oriented isotropically. In a more recent study by Latawiec et al., D–Bχ–A dyads were synthesized, in which Bχ corresponds to a B-form DNA helix composed of 4–6 base pairs.^12^ Also in this case the TREPR spectra of these SCRPs show that a significant contribution from the CISS effect is required to reproduce the experimental observations.

Together, these studies provided direct evidence of the CISS effect in chiral D–Bχ–A dyads, where electrons travel through a chiral bridge in molecules dispersed in solution. The absence of any substrate in these experiments supports theories that attribute the entire CISS effect to chiral organic molecules.^13,17–19^ Reconciling the high efficiency of CISS with the low spin–orbit coupling of the light atoms in the investigated organic molecules has been a challenge for proposed theories of CISS. Explaining the observed polarizations requires additional ingredients such as electron–electron correlations.^20,21^

Building on these recent advances in the investigation of CISS in intramolecular processes, the next conceptual step is to investigate systems in which the handed moiety is the donor unit rather than the bridge. Such systems would allow understanding of whether the CISS effect arises from electron transfer through a chiral bridge or is an intrinsic property of the overall chiral molecule. To this end, we designed new chiral donor–acceptor (Dχ–B–A) dyads incorporating a thia-bridged[4]­helicene as the donor. Whereas helicenes^22–26^ are often encountered in CISS experiments,^27,28^ we selected thiahelicenes^29,30^ for their favorable redox properties, which allow the isolation and processing of the cationic form.^31–33^ Additionally, previous transport measurements revealed high CISS efficiency at low voltages for both the neutral and cationic forms.^34,35^ This donor was covalently linked, via an appropriate bridging moiety comprising three *p*-phenyleneethynylene units, to a perylene-3,4:9,10-bis­(dicarboximide) (PDI) acceptor. For comparison, an achiral model reference was synthesized, featuring an *N-*aryl phenothiazine donor lacking one sulfur bridge that confers chirality, linked to the same PDI acceptor. Upon selective photoexcitation of PDI, both the chiral and achiral dyads undergo a hole transfer process leading to the formation of an SCRP (Dχ^·+^–B–A^·–^), whose spin features were investigated by TREPR spectroscopy.

To the best of our knowledge, these systems represent the first example of chiral donor–acceptor dyads incorporating helicenes, as well as the first spin-correlated radical pair based on a helicene framework. Despite the pronounced spin-filtering capabilities of dithia-aza[4]­helicenes when anchored on a surface and subjected to an electric current, present findings suggest their use as chiral donor units results in a weaker CISS contribution than that observed in donor–acceptor dyads featuring a chiral bridge. These findings represent a step toward a more comprehensive theoretical understanding of spin selectivity in molecular systems and contribute to establishing a rational design of CISS-active donor–acceptor dyads.

## Results and Discussion

### Dyads Engineering and Synthesis

We engineered our donor–acceptor dyads with the PDI and dithia-aza[4]­helicene units acting as acceptor and donor, respectively (Figure 1a). PDI and its derivatives are a well-known class of compounds, characterized by low reduction potential, strong absorption in the visible region, high fluorescence quantum yield in dilute solutions, and high molar extinction coefficients.^36–41^ Dithia-aza[4]­helicenes are a rare example of enantiomerically stable hetero[4]­helicenes, owing to the presence of four long carbon–sulfur bonds, which induce a pronounced overlap of the terminal rings and lead to high racemization barriers.^29,30,42^ Moreover, these compounds are particularly fascinating due to their redox properties, as they can be easily oxidized to their corresponding radical cations through a reversible reaction.^31,32^ This makes them excellent candidates as a chiral donor in donor–acceptor dyads. Furthermore, cyclic voltammetry measurements (see Supporting Information for details) on the donor–acceptor dyads confirmed suitable redox potentials for efficient electron transfer to occur.

**Figure 1:**
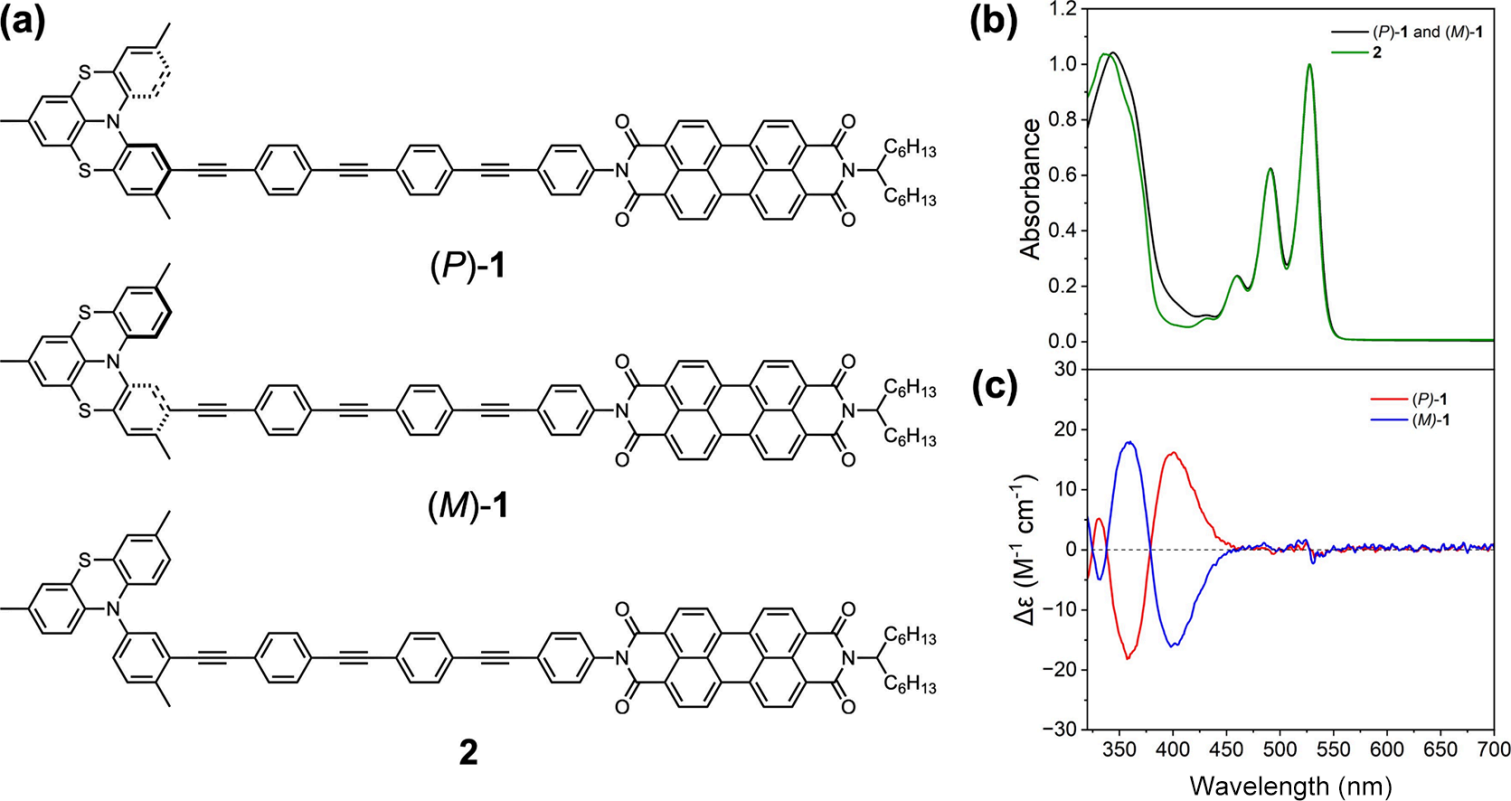
(a) Structure of chiral dyads (*P*)-**1** and (*M*)-**1** and the achiral model reference.^2^ (b) UV–vis absorption spectra of (*P*)-**1**, (*M*)-**1**, and **2** recorded in toluene at room temperature. (c) CD spectra of (*P*)-**1** and (*M*)-**1** recorded in DCM at room temperature. See Figure S16 for corresponding computed spectra.

The bridge unit was synthesized via a Sonogashira cross-coupling reaction starting from 4-iodophenol and trimethylsilylacetylene (TMSA). The achiral donor unit, consisting of an N-arylphenothiazine, and the thia-bridged[4]­helicene chiral unit, obtained by reaction with phthalimidesulfenyl chloride, were prepared according to a previous synthetic procedure.^29^ On both achiral and chiral donors a triflate group was installed and coupled with the terminal alkyne of the bridge to afford the donor-bridge units. The perylene monoimide monoanhydride (PMI) was obtained in two steps starting from perylene-3,4,9,10-tetracarboxylic dianhydride. A branched alkyl chain containing 13 carbon atoms was attached to one of the nitrogen positions to enhance the solubility of the final system. Finally, the racemic chiral (**rac1**) and achiral (**2**) donor–acceptor dyads were synthesized through the reaction of PMI with the donor-bridge units in the presence of imidazole. Enantiopure (*P*)-**1** and (*M*)-**1** were obtained by chiral HPLC resolution. Synthetic details are provided in the Supporting Information.

### Absorption and Circular Dichroism (CD) Spectroscopy

The ultraviolet–visible (UV–vis) absorption spectra of (*P*)-**1** and (*M*)-**1** differ from that of **2** only for a slightly weaker absorbance at wavelengths <400 nm (Figure 1b). The spectra are dominated by the PDI absorption bands at 527, 491, and 459 nm. Circular dichroism (CD) spectra of each enantiomer show the expected mirror image symmetry (Figure 1c), with the CD response dominated by the helicene unit and only negligible contributions from the PDI. The dissymmetry factor *g*_dis_ = 2­(ε_L_ – ε_R_)/(ε_L_ + ε_R_) (see Figure S14), often employed to monitor chirality transfer,^43^ is practically zero over the PDI region. This suggests that the use of a rigid long linker has successfully localized the chirality on the donor unit.

### Excited-State Dynamics

The charge transfer and recombination dynamics of dyad **rac1** were investigated in 2-MeTHF at 85 K using transient absorption (TA) spectroscopy (Figure 2a–d). The TA measurements were collected over a spectral range of 380–1600 nm. The time windows were 7 ns with ca. 300 fs time resolution and 300 μs with 0.6 ns resolution. Evolution-associated spectra (EAS) obtained from global fitting of the combined data sets, along with the corresponding population dynamics based on a sequential kinetic model, are shown in Figure S16. Upon selective photoexcitation of PDI at 530 nm, the TA spectrum exhibits the characteristic features of the singlet excited state ^1*^PDI.^44,45^ Ground-state bleaching (GSB) is observed between 440 and 560 nm, stimulated emission (SE) appears at 580 and 625 nm, and broad excited-state absorption (ESA) from S_n_ ← S_1_ extends from 630 to 1100 nm. After a few nanoseconds, a distinct ESA feature emerges at 950 nm, assigned to the PDI^·–^ radical anion,^46^ together with a broad band above 1000 nm, assigned to the thiahelicene radical cation, indicative of charge transfer (CT) state formation.^47^

**Figure 2:**
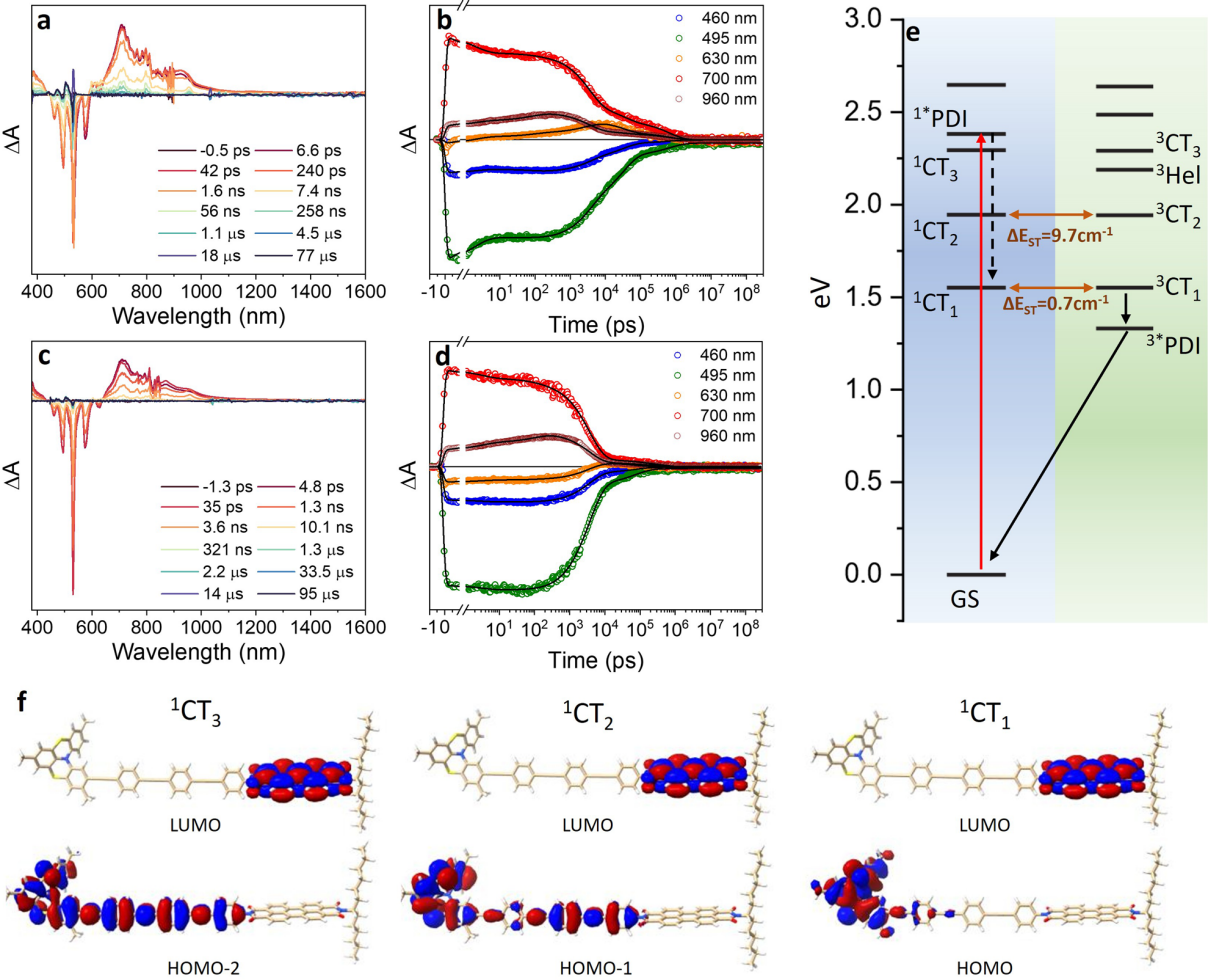
TA spectra of dyads **rac1** (a) and **2** (c) in 2-MeTHF at 85 K, excited at 530 nm (100 fs, 0.5 μJ/pulse), recorded at selected pump–probe delays. (b, d) Kinetic traces at representative wavelengths with corresponding global fits. (e) Energy level diagram of **1** calculated via DFT, with arrows indicating the photophysical pathways based on the combined TA and DFT analysis. Singlet and triplet states are reported on the left and right sides of panel e, respectively. (f) Molecular orbital contributions to the electronic transitions of CT_1–3._

DFT calculations indicate the presence of three energetically accessible CT states (^1^CT_3_,^1^CT_2_,^1^CT_1_), which may relax sequentially (Figure 2e). Analysis of the computed transitions shows that these states share a very similar electronic character (Figure 2f), all arising from electron transfer from helicene-based HOMO orbitals to the PDI LUMO. In particular, the HOMOs of ^1^CT_2_ and ^1^CT_3_ display mixed helicene/bridge character, whereas ^1^CT_1_ exhibits complete HOMO localization on the helicene donor. Conversely, the LUMO of all three CT states is fully localized on the PDI acceptor. This suggests that ^1^CT_2_ and ^1^CT_3_ may act as intermediate CT states that subsequently relax to the fully charge-separated ^1^CT_1_ state. As expected, the DFT calculations also show that, for each singlet CT state, a triplet CT state lies close in energy. In the TA spectrum, the CT state signatures persist for several hundred nanoseconds, gradually evolving into the ^3*^PDI triplet state, characterized by ESA bands between 420 and 530 nm, as the charge transfer state recombines. DFT calculations are consistent with this assignment as they reveal a low-lying excited triplet state localized on the PDI (Figure 2e).

Based on the TA features and the DFT calculations, the global fitting was performed using the kinetic model A → B → C → D → E → F. States A, B, and C correspond to the singlet excited state of PDI, which relaxes and undergoes hole transfer with multiple time constants due to distributed kinetics in the low-temperature matrix. State D emerges with τ_CT_ = 3.32 ± 0.05 ns and is assigned to the CT state. Notably, it is not possible to determine whether this EAS corresponds to the intermediate CT_2–3_ states or already to the lower-lying CT_1_, as their electronic characters - and consequently their spectral signatures - are very similar. State E exhibits an EAS closely resembling that of D, suggesting either a relaxed form of the CT state (i.e., CT_1_) or additional contributions from distributed kinetics. Finally, the CT_1_ state recombines with τ_CR_ = 664.1 ± 0.6 ns, leading to state F, which is attributed to the PDI triplet state.

The TA spectra of the achiral reference dyad at 85 K in 2-MeTHF are shown in Figures 2 and S17. The spectra and kinetics closely resemble those of **rac1**, with only minor differences in the time constants. The same sequential kinetic model was applied for the global analysis. As in the chiral case, photoexcitation of PDI leads to the formation of the singlet excited state ^1*^PDI, followed by hole transfer with a time constant of τ_CT_ = 3.60 ± 0.03 ns, in very good agreement with the chiral counterpart. From there, charge recombination to ^3*^PDI occurs with τ_CR_ = 431.1 ± 0.6 ns.

### Time-Resolved EPR

The X-band TREPR spectra of dyads **rac1** and **2** were recorded in frozen toluene solution at 85 K, following photoexcitation at 530 nm (Figure S21). Spectra were acquired in direct detection mode, where positive signals indicate enhanced absorption (*a*) and negative signals correspond to emission (*e*).^48^ Spectra of both samples display an *ea* polarization pattern that is attributed to a radical pair.^10,11,49,50^ The narrow central line is the PDI radical anion,^51^ whereas the broader shoulders arise from the thiahelicene radical cation, which exhibits anisotropic *g*- and hyperfine tensors.^31,33^ A preliminary comparison of the spectra reveals differences in the relative intensity of the central peak with respect to the shoulders.

To further investigate these effects and the anisotropic nature of the radical pair, orientation-dependent TREPR measurements were performed. To do this, the samples were dissolved in the nematic liquid crystal 4-cyano-4′-(*n*-pentyl)­biphenyl (5CB), aligned in a magnetic field at 295 K, and rapidly frozen to 85 K. This procedure aligns the long molecular axes of 5CB along the magnetic field direction, which in turn, aligns the cylindrically symmetric dyads in the same direction and allows the frozen sample to be rotated relative to the magnetic field. In the following, we discuss spectra acquired with the 5CB molecules oriented either parallel or perpendicular to the magnetic field (Figure 3a). TREPR spectra of dyads **rac1** and **2** in 5CB at 85 K, after photoexcitation at 530 nm, were recorded using both a wide (120 mT, Figure S22) and a narrow (15 mT, Figure S23) field sweep to probe the photoexcited triplets and radical pairs, respectively.

**Figure 3:**
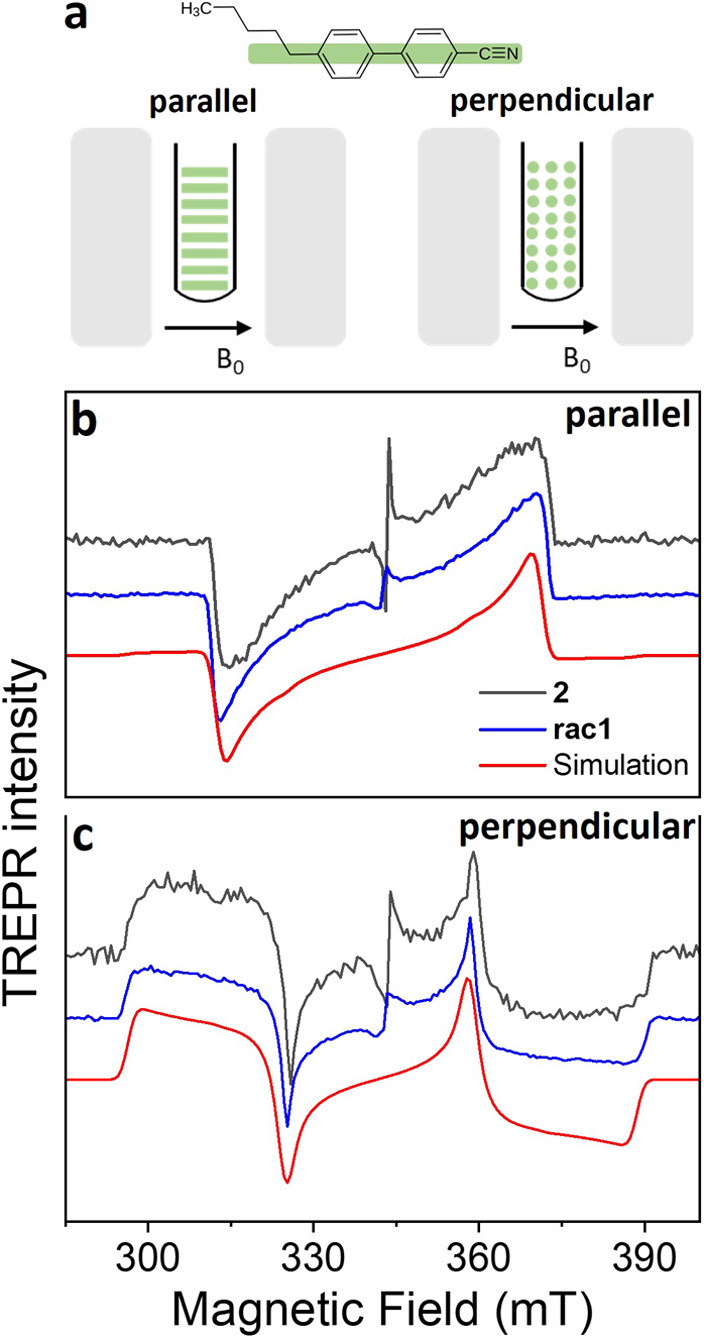
(a) Schematic illustration of the 5CB liquid crystal alignment in the two experimental orientations. (b, c) Normalized 1D experimental TREPR spectra of dyads **rac1** (blue) and **2** (black) dissolved in the nematic liquid crystal 5CB at 85 K. The spectra were recorded 1 μs after a 530 nm unpolarized laser pulse (7 ns, 2 mJ; integration window = 200 ns). Measurements were performed with the liquid crystal oriented parallel (b) and perpendicular (c) to the magnetic field, corresponding to dyads whose long molecular axis (*y’*-axis in Figure 4a) is parallel or perpendicular to B_0_. The broad spectral features spanning 290–390 mT are attributed to the PDI excited triplet state and are simulated (red line) using the parameters listed in Table S1.

In Figure 3b/c, the spectra obtained 1 μs after unpolarized laser excitation show orientation-dependent signals characteristic of photoexcited triplet states.^52,53^ Spectral simulations, with parameters reported in Table S1, yielded zero-field splitting values of *D* = 1300 MHz and *E* = −120 MHz, providing insight into wave function delocalization and supporting the assignment to the PDI triplet.^54,55^ The relative triplet sublevel (*T′*_–1_, *T′*_0_, *T′*_+1_) populations give information about the triplet generation mechanism.^56,57^ Specifically, the simulated polarization pattern corresponds to neutral triplet states resulting from radical pair recombination, with preferential population of the high-field *T′*_0_ sublevel (normalized populations: *p′*_–1_ = 0, *p′*_0_ = 1, *p′*_+1_ = 0). This polarization pattern is characteristic of triplets formed via radical pair intermediates, where spin-allowed decay of the *T*_0_ radical pair sublevel selectively populates the *T′*_0_ sublevel of the triplet state.^54,55,58^ Importantly, the triplet simulations serve as an internal confirmation of the dyad orientation in 5CB. Both chiral and achiral spectra can be reproduced using the same orientational distribution of the molecules in the sample. Referring to Figure 4a, in the parallel case, the *y*′**-axis of the PDI triplet (nearly collinear with the bridge) lies parallel to the magnetic field. In contrast, in the perpendicular case, the *y*′**-axis is orthogonal to the field, and the *x*′*z*′**-plane of the triplet is probed.

**Figure 4:**
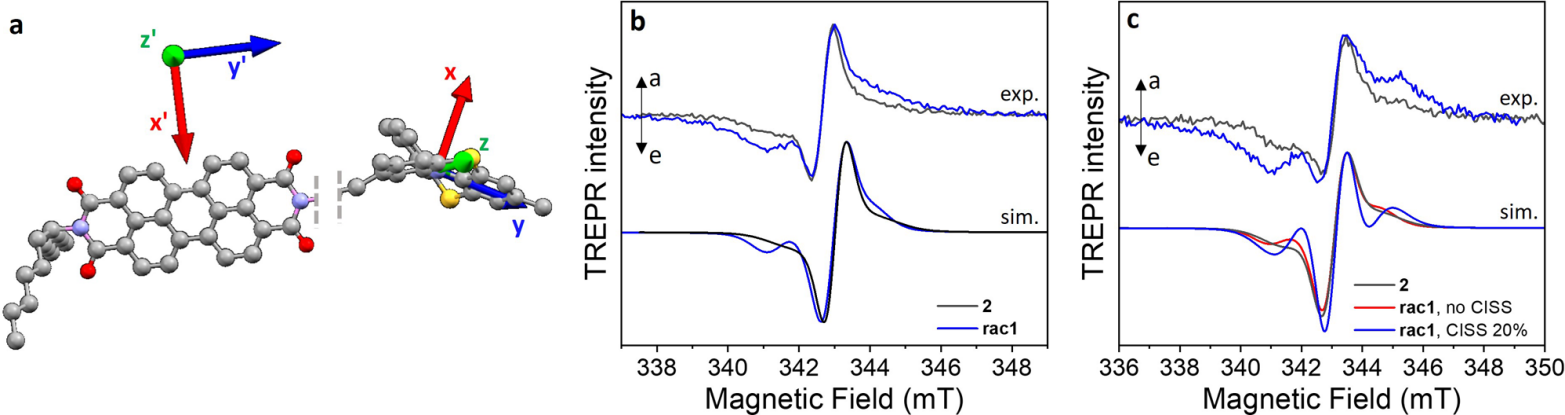
(a) Representation of the relative orientation between the reference frame of the PDI (defined by the triplet zero-field splitting tensor) and that of the thiahelicene (defined by the g-tensor of the radical cation). In this reference frame, the A-tensor is (18.24, −15.88, −10.54; −15.88, 32.78, 16.47; −10.54, 16.47, 19.05). The axial dipolar interaction is along the line connecting the two radical centers, which is nearly collinear with the *y*′-axis. (b, c) Normalized 1D experimental TREPR spectra of **2** (black) and **rac1** (blue) dyads, recorded 400 ns after the unpolarized laser pulse (integration window = 100 ns). Dyad **rac1** and **2** aligned in the nematic liquid crystal 5CB at 85 K following a 530 nm unpolarized laser pulse (7 ns, 2 mJ). Spectra were measured with the liquid crystals oriented parallel (b) and perpendicular (c) to the magnetic field. The corresponding simulations, performed using the parameters defined in the text for both chiral and achiral variants, are also shown. The occurrence of CISS does not alter the spectrum of dyad **rac1** in the parallel orientation (b), whereas in the perpendicular orientation (c) it enhances the lateral shoulders, in agreement with the experimental observations.

With this orientation established, we now turn to the narrow field sweep. Figure S23 shows the full 2D contour plots, while Figures 4b/c present spectra obtained 400 ns after the unpolarized laser pulse for dyad **rac1** and **2**, together with their corresponding simulations. In both cases, and for both orientations, an *ea* signal characteristic of a radical pair is observed, similar to that seen in isotropically oriented toluene. A closer inspection (Figure S24) reveals a larger change in the intensity of the shoulders at 341 and 346 mT when going from parallel to perpendicular in **rac1** compared to **2**.

The detected spin polarization arises only when CT_1_ is formed; in the initial charge transfer states, where the hole is delocalized also on the bridge, the exchange interaction between the two spins is too strong to allow significant spin evolution from the singlet-born radical-pair state to the triplet radical-pair state.^49^ When the CISS effect is present, depositing the spin on the chiral helicene moiety in the final radical pair induces an additional triplet character, which is reflected in the spin-polarized TREPR spectra.

To quantify a possible CISS contribution, we simulated the TREPR signals of dyads **rac1** and **2** (Figure 4b,c). The spin Hamiltonian includes the contributions of the two radical ions (*H*_1_ and *H*_2_) as well as an interaction term comprising an axial dipole–dipole coupling along the direction of the spin centers - evaluated within the point-dipole approximation using an interspin separation of 3.04 nm - and an isotropic exchange *J* **S**_1_ · **S**_2_. The Hamiltonian of the PDI radical anion is given by a Zeeman interaction with the external magnetic field **B**: *H*_1_ = *g*_1_μ_B_ **B**·**S**_1_, where the isotropic *g*-value is known from previous studies, *g*_1_ = 2.004.^54^ For the radical cation, the Hamiltonian is

$$H_{2}=\mu_{B}B\cdot g_{2}\cdot S_{2}+I_{N}\cdot A_{N}\cdot S_{2}$$ 1

where **g**_2_ is the anisotropic g*-*tensor of the cation and **A**_N_ the hyperfine coupling tensor describing the interaction of the electron spin *S*_2_ with the nuclear spin *I*_N_ = 1 of the nearby nitrogen atom. Because these parameters are not available in the literature, we determined them through DFT calculations on the donor radical cations, namely dithia-aza[4]­helicene for **rac1** and N-arylphenothiazine for **2** (Table S2). To validate these values, we synthesized the corresponding model derivatives containing chiral and achiral donor units and generated their radical cations by chemical oxidation in DCM (see the SI for synthetic details). The echo-detected EPR (EDEPR) spectra of these radicals, together with their simulations (Figures S24 and S25), confirm the accuracy of the computed parameters.

Starting from these values, we have performed a fit of the spectra of the chiral and achiral dyads, for the two orientations of the molecules with respect to the applied field (Figure 3b,c). In particular, we have kept fixed the orientation of the tensors derived *ab initio*, see Figure 4a for **g**_2_, and we have varied only the principal values. Guided again by DFT, we have kept the same principal values of **A**_N_ = (20, 40, 30) MHz for both chiral and achiral molecules, and only slightly varied **g**_2_, namely (2.0040, 2.0047, 2.0069) for the chiral and (2.0041, 2.0042, 2.0069) for the achiral dyad. As for the spin–spin interaction between the two radicals, the recorded spectra display the same *ea* character for both parallel and perpendicular orientations. For a singlet state precursor, as in the achiral dyad, this implies a predominant ferromagnetic interaction in both orientations, corresponding to a lower bound of approximately 2 MHz for the exchange coupling.

CISS manifests in a triplet component in the spin state of the SCRP,^10–12,19^ which can be represented as^59^

$$\left|\psi \rangle =\cos\;\frac{\chi }{2}|S\rangle +i\;\sin\;\frac{\chi }{2}|T_{0}(\theta )\right\rangle$$ 2

where |*S*⟩ and |*T*_0_(θ)⟩ are the singlet and triplet (*m* = 0) components, respectively. The latter depends on the angle θ between the external field and the chiral axis. In the present case, the angle between the chiral axis on the helicene donor and the dipolar axis is about 60°, as derived from DFT optimization of the molecular structure. This is kept fixed during the spectral simulation.

The angle χ in eq 2 is equal to 0 for a singlet precursor (no CISS, as in the achiral dyad) and is π/2 for maximum CISS effect. Note that EPR is sensitive to the triplet population and not to the relative phase between |*S*⟩ and |*T*_0_(θ)⟩, which can also be real. Hence, in the following, we quantify CISS by introducing the CISS efficiency $p_{\mathrm{CISS}}=2\sin^{2}\;\frac{\chi }{2}$ , which is similarly bounded between 0 (χ = 0) and 1 (χ = ±π/2). The presence of CISS cannot be inferred from the spectrum measured in the parallel orientation, as the CISS and no-CISS cases yield indistinguishable spectral features.^19^ Conversely, our simulations show that in the perpendicular orientation a CISS contribution (χ ≠ 0) leads to more pronounced external shoulders as observed in the data. In particular, in Figure 3, a CISS efficiency *p*_CISS_ = 0.2 is assumed. Note that other factors could also influence the differences observed in the spectra. For instance, amplification of the external shoulders can be reproduced by introducing larger modifications to the system Hamiltonian - such as additional hyperfine couplings, variations in the orientation of the **A**_N_ tensor. Conversely, the smoothing of the shoulders may arise from orientational disorder and/or larger values of *J*. Overall, within the model used to simulate the data, a value of *p*_CISS_ = 0.2 should be regarded as an upper bound for the CISS-induced spin polarization.

## Conclusions

In this study, we reported the synthesis and spin-photophysical characterization of a new donor–acceptor dyad comprising a thiahelicene donor and a PDI acceptor linked by a tris­(*p*-phenyleneethynylene) bridge. Upon selective photoexcitation of the PDI at 85 K, hole transfer occurs, generating a long-lived (>400 ns) radical pair that subsequently undergoes charge recombination to form a PDI triplet. Both photoexcited paramagnetic species are observed by TREPR. This dyad, therefore, provides an ideal platform to investigate spin polarization induced by the CISS effect at the molecular level when the handed portion is the donor, rather than the electron-transfer bridge.

The well-established orientation dependence of TREPR spectra of the PDI triplet allowed us to assess the molecular alignment of the dyad in the liquid-crystal solutions. With the molecular orientation thus established, spectral simulations of the radical pair were performed. Overall, simulations for two representative molecular orientations relative to the external magnetic field indicate a rather small CISS contribution. The estimated CISS efficiency of about 20% is significantly smaller than that detected in spin-transport by mc-AFM measurements on related thiahelicenes, which is above 50% in the neutral^34^ and around 60% in the radical cation forms.^34^ Notably, previous TREPR studies on D-chiral bridge-A systems revealed efficiencies up to ∼50%,^10^ comparable to what was obtained from transport experiments on the same chiral linker.^10,16^

Several factors can contribute to the observed difference. The most relevant one is the position of the chiral moiety inside the dyad. The current understanding of the CISS phenomenon^17,60^ suggests that the spin polarization at the extremes of the dyad arises from an interplay between incoherent electron motion from the donor to the chiral bridge and then to the acceptor, and coherent dynamics on the bridge. Our findings suggest that while CISS in transport measurements is commonly observed, its occurrence in photoinduced ET requires more stringent molecular design. According to existing theories, a partial reduction of CISS efficiency might also be induced by the noncollinearity between the chirality axis and the electron displacement vector.^61–63^ Experimental studies have addressed geometrical aspects,^64,65^ and an mc-AFM platform has been specifically designed to investigate the direction dependence. The investigation of CISS at the intramolecular level allows, through chemical design, modifications of the angle between the chirality vector and the ET direction, as well as easy control of the orientation of the external magnetic field, thereby directly assessing the role of these geometric factors. In addition, exploring different exchange-coupling regimes - where the balance between anisotropic (dipolar) and isotropic spin–spin interactions within the radical pair is varied - will be crucial. A more accurate quantification of CISS could be achieved by investigating systems with fully poled dyads, i.e., systems in which all donor–acceptor molecules share not only the same parallel orientation, as in our 5CB measurements, but also are unidirectionally aligned with respect to the external magnetic field. Under such conditions, the two enantiomers give distinguishable TREPR spectra if CISS is active, and its efficiency can be quantified as the difference spectrum between the enantiomers, as shown in Figure S26.

Overall, our findings add another piece to the puzzle of the molecular origin of the CISS effect and point to two key molecular design principles: the spatial localization of chirality and the relative orientation of the chiral axis with respect to the ET direction as critical parameters governing CISS efficiency.

## Supplementary Material



## Notes

Materials and synthesis, CD spectra, cyclic voltammetry, additional transient absorption spectra, computational details, additional TREPR spectra, and simulations (PDF)
