# Supplementary material for: The Role of Chirality-Induced Spin Selectivity in Helicene-Based Photogenerated Radical Pairs
Source: J Am Chem Soc. 2026 Jun 13;148(25):25623–31. doi: 10.1021/jacs.6c02634 (PMC13339141; doi:10.1021/jacs.6c02634)
Supplement: Supplementary file 1 [file ja6c02634_si_001.pdf]

Supplementary Information for

The Role of Chirality-Induced Spin Selectivity in Helicene-Based Photogenerated Radical Pairs

Giulia Agnoloni[1,2], Alessandro Chiesa,[3,4] Ryan M. Young,[2] Federico Totti,[1] Stefano Menichetti,[1] Caterina Viglianisi,[1*] Michael R. Wasielewski,[2*] Stefano Carretta,[3*] Alberto Privitera,[2,5*] and Roberta Sessoli[1*]

[1] *Department of Chemistry "U. Schiff", University of Florence & UdR INSTM Firenze, 50019, Sesto Fiorentino, Italy*

[2] *Department of Chemistry, Center for Molecular Quantum Transduction, and Institute for Quantum Information Research and Engineering, Northwestern University, 60208-3113, Evanston, IL (USA)*

[3] *Department of Mathematical, Physical and Computer Sciences, University of Parma & UdR INSTM, 43124, Parma, Italy*

[4] *INFN-Sezione di Milano Bicocca, Gruppo Collegato di Parma, 43124 Parma, Italy*

[5] *Department of Industrial Engineering, University of Florence & UdR INSTM Firenze, 50139, Firenze, Italy*

Table of Contents

1. Materials, synthesis, and characterization 3

2. HPLC and Circular Dichroism Spectroscopy 14

3. Cyclic Voltammetry 16

4. Transient absorption spectroscopy 18

5. Computational details 21

6. Electron Paramagnetic Resonance 25

7. References 33

1. Materials, synthesis, and characterization

Materials. ^{1}H and ^{13}C NMR spectra were recorded with *Varian MercuryPlus 400* and *Varian Inova 400*, using $CDCl_3$ and $(CD_3)_2CO$ as solvents. Residual $CHCl_3$ at δ = 7.26 ppm and CD_3COCD_2H at δ = 2.05 ppm were used as internal references for ^{1}H NMR spectra; central line of $CDCl_3$ at δ = 77.16 ppm and $(CD_3)_2CO$ at δ = 30.60 were used as internal references for ^{13}C NMR spectra. Fourier-transformed infrared (FT-IR) spectra were recorded with a *PerkinElmer SpectrumTwo* spectrometer. Electrospray ionization mass spectra (ESI-MS) were recorded with *LC-MS LCQ Fleet ThermoFisher Scientific* instrument. Melting points were measured with a *Stuart SMP50 Automatic Melting Point Apparatus*. Thin-layer chromatography (TLC) on commercially available precoated plates (silica gel 60 F_{254}) were used to monitor the reactions, and the products were visualized with acidic vanillin solution. Column chromatography was performed using Silica gel 60 (230–400 mesh). Dry solvents were obtained from a *PureSolv Micro Solvent Purification System*. Elemental analyses were performed on a *ThermoScientific FlashSmart CHNS/O Analyzer*.

Synthesis and characterization. The synthesis and characterization of all the donor-acceptor dyads are described in detail below. All chemicals were obtained from commercial suppliers and used as received without further purification. $Pd(PPh_3)Cl_2$, $Pd(PPh_3)_4$, and triflic anhydride were stored under nitrogen; triethylamine (TEA) and pyridine were freshly distilled over KOH before use.

Synthesis of the acceptor unit

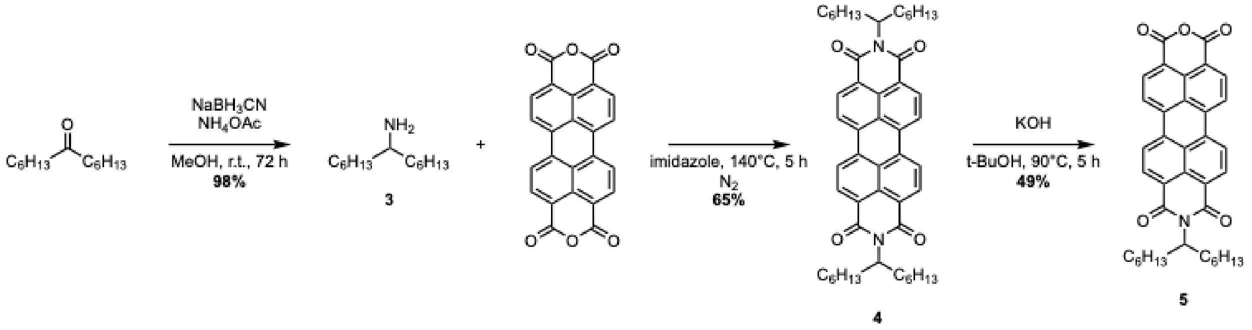

Figure S1. Synthesis of the perylene monoimide (**PMI) 5**.

Tridecyl-7-amine (3). A round-bottom flask was charged with di-*n*-hexylketone (900 mg, 4.55 mmol, 1 eq), NH_4OAc (3.5 g, 45.5 mmol, 10 eq), $NaBH_3CN$ (315 mg, 5 mmol, 1.1 eq), and

methanol (15 mL). The reaction mixture was stirred at room temperature for 72 h. At the end, HCl 12 M (~ 0.6 mL) was added dropwise. The solvent was removed via rotary evaporation vacuum. The solid was diluted with H_2O (100 mL), taken to pH 10 with KOH, and the organic phase extracted with $CHCl_3$ (2x40 mL). The organic layer was collected, dried over Na_2SO_4, filtered, and the volatiles were removed via rotary evaporation. The crude was used for the next steps without further purification (889 mg, 98% yield).

PDI (4). A round-bottom flask was charged with PTCDA (500 mg, 1.28 mmol, 1eq), **3** (625 mg, 3.14 mmol, 2.45 eq), and imidazole (2 g, 30 mmol, 12 eq). The reaction mixture was stirred at 150°C for 5 h, under the N_2 atmosphere. Once cooled to room temperature, ethanol (15 mL) and HCl 2N (55 mL) were then added and the mixture was stirred overnight. The red precipitate was then collected through the Büchner filter, rinsed thoroughly with H_2O, and dried to 100°C, to give the **4** (966 mg) as a red solid (65%). **^{1}H NMR** (400 MHz, $CDCl_3$, δ): 8.67-8.61 (m, 8H), 5.22-5.15 (m, ,2H), 2.29-2.20 (m, 4H), 1.91-1.82 (m, 4H), 1.39-1.23 (m, 32 H), 0.82 (t, J=8 Hz, 12 H), ppm coincides with the literature.[1]

PMI (5). A round-bottom flask was charged with **4** (290 mg, 0.384 mmol, 1 eq) and t-BuOH (10 mL). KOH powder (1.1 g, 1.92 mmol, 5 eq) was then added. The reaction mixture was stirred at 90°C for 5 h, under the N_2 atmosphere. Once cooled to room temperature, acetic acid (9 mL) was added slowly with a vigorous stirring, for 15 h. Then HCl 2N (5 mL) was added and stirred for another 30 minutes. The resulting precipitate was collected through the Büchner filter, and rinsed thoroughly with H_2O, until pH 7. The red solid was then dried at 100°C. The crude was purified by flash chromatography on silica gel with $CHCl_3$ to obtain **5** (108 mg) as a red solid (49% yield). **^{1}H NMR** (200 MHz, $CDCl_3$, δ): 8.73-8.63 (m, 8H), 5.25-5.11 (m, 1H), 2.31-2.19 (m, 2H), 1.92-1.80 (m, 2H), 1.30-1.22 (m, 16 H), 0.82 (t, J = 8 Hz, 6 H), ppm coincides with the literature.[1]

Synthesis of the bridge

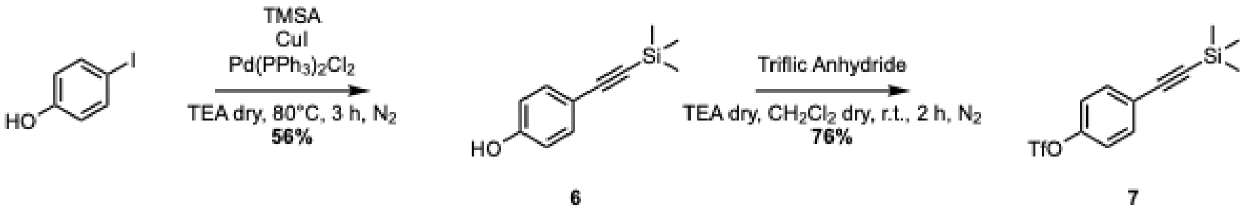

6 7

Figure S2. Synthesis of 4-((trimethylsilyl)ethynyl)phenyl trifluoromethanesulfonate (**7**).

4-((trimethylsilyl)ethynyl)phenol (6). A dry Schlenk tube was charged with 4-iodophenol (1 g, 4.5 mmol, 1 eq), $Pd(PPh_3)Cl_2$ (97 mg, 0.14 mmol, 0.03 eq), CuI (26 mg, 0.14 mmol, 0.03 eq). Three vacuum-nitrogen cycles were performed, and through syringe dry and degassed triethylamine (11 g, 15 mL, 108 mmol, 24 eq), and trimethylsilyl acetylene (TMSA, 658 mg, 0.95 mL, 6.7 mmol, 1.5 eq) were added. The reaction mixture was stirred under nitrogen flux at 80°C for 3 h. Once cooled to room temperature, the solid was removed through filtration and the solution was collected and the volatiles removed via rotary evaporation. The crude product was purified by flash chromatography on silica gel with hexane : ethyl acetate (2 : 1), to afford the product **6** as white crystals (478 mg, 56%). **^{1}H NMR** (200 MHz, $CDCl_3$, δ): 7.39-7.34 (m, 2H), 6.77-6.73 (m, 2H), 0.23 (s, 9H), ppm coincides with the literature.[2]

4-((trimethylsilyl)ethynyl)phenyl trifluoromethanesulfonate (7). A round-bottom flask was charged with **6** (237 mg, 1.25 mmol, 1 eq) and dry CH_2Cl_2 (1.6 mL). The resulting solution was cooled to 0°C, and then added dry triethylamine dropwise (255 mg, 0.4 mL, 2.5 mmol, 2 eq). A solution of triflic anhydride (422 mg, 252 µL, 1.5 mmol, 1.2 eq) in dry CH_2Cl_2 (0.520 mL) was then added dropwise at 0°C. The reaction mixture was stirred under the N_2 atmosphere at room temperature for 17 h. The mixture was diluted with diethyl ether (75 mL) and washed with NH_4Cl (2 x 50 mL), $NaHCO_3$ (2 x 50 mL), H_2O (2 x 50 mL) and brine (50 mL). The crude was purified by flash chromatography on silica gel with hexane : ethyl acetate (15 : 1), to afford the product **7** (305 mg) with a yield of 76%. **^{1}H NMR** (200 MHz, $CDCl_3$, δ): 7.55-7.51 (m, 2H), 7.24-7.19 (m, 2H), 0.25 (s, 9H), ppm coincides with the literature.[3]

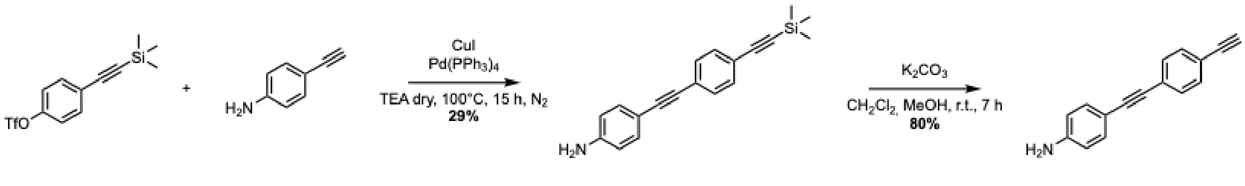

Figure S3. Synthesis of 4-((4-ethynylphenyl)ethynyl)aniline (**9**).

4-((4-((trimethylsilyl)ethynyl)phenyl)ethynyl)aniline (8). A dry Schlenk tube was charged with **7** (292 mg, 0.906 mmol, 1 eq), 4-ethynilaniline (248 mg, 1.4 mmol, 1.5 eq), $Pd(PPh_3)_4$ (52 mg, 0.045 mmol, 0.05 eq), CuI (9 mg, 0.045 mmol, 0.05 eq). Three vacuum-nitrogen cycles were performed, and through syringe dry and degassed triethylamine (2.5 g, 3.5 mL, 25.4 mmol, 28 eq) was added. The reaction mixture was stirred under nitrogen flux at 90°C for 17 h. Once cooled to

room temperature, the mixture was diluted with $CHCl_3$ (50 mL) and washed with H_2O (2 x 50 mL). The organic layer was collected and the volatiles removed via rotary evaporation. The crude product was purified by flash chromatography on silica gel with CH_2Cl_2, to afford the product **8** (76 mg) with a yield of 29%. **^{1}H NMR** (400 MHz, $CDCl_3$, δ): 7.41 (s, 4 H), 7.34-7.32 (m, 2H), 6.65-6.62 (m, 2H), 3.84 (s, 2 H), 0.25 (s, 9H), ppm coincides with the literature.[4]

4-((4-ethynylphenyl)ethynyl)aniline (9). A round-bottom flask was charged with **8** (128 mg, 0.44 mmol, 1 eq), K_2CO_3 (306 mg, 2.21 mmol, 5 eq), MeOH (3 mL), and CH_2Cl_2 (3 mL). The reaction mixture was stirred under nitrogen atmosphere at room temperature for 7 h. The solid was removed through filtration and washed with methanol. The solution was collected and the volatiles removed via rotary evaporation. Without further purification, compound **9** (77 mg) was obtained with a yield of 80%. **^{1}H NMR** (200 MHz, $CDCl_3$, δ): 7.44 (s, 4H), 7.36-7.31 (m, 2H), 6.66-6.62 (m, 2H), 3.84 (s, 2H), 3.16 (s, 1H) ppm coincides with the literature.[5]

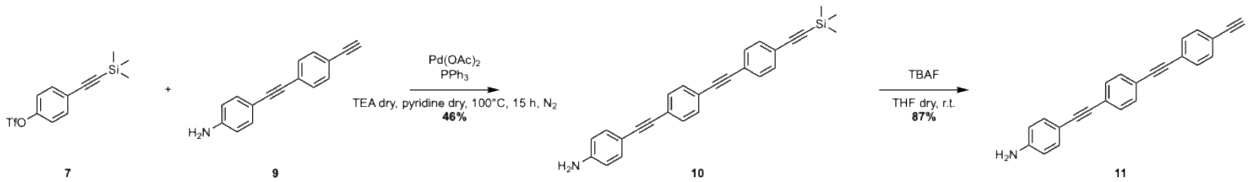

Figure S4. Synthesis of 4-((4-((4-ethynylphenyl)ethynyl)phenyl)ethynyl)aniline (**11**).

4-((4-((4-((trimethylsilyl)ethynyl)phenyl)ethynyl)phenyl)ethynyl)aniline (10). A dry Schlenk tube was charged with **7** (305 mg, 0.95 mmol, 1 eq), **9** (304 mg, 1.4 mmol, 1.5 eq), PPh_3 (324 mg, 1.24 mmol, 1.3 eq), $Pd(OAc)_2$ (11 mg, 0.048 mmol, 0.05 eq). Three vacuum-nitrogen cycles were performed, and through syringe dry and degassed triethylamine (20.3 g, 28 mL, 201 mmol, 212 eq) and pyridine (2.7 g, 2.8 mL, 35 mmol, 37 eq) were added. The reaction mixture was stirred under nitrogen flux at 90°C for 17 h. Once cooled to room temperature, the mixture was diluted with CH_2Cl_2 (250 mL) and washed with H_2O (2 x 100 mL). The organic layer was collected and the volatiles removed via rotary evaporation. The crude product was purified by flash chromatography on silica gel with hexane : ethyl acetate (3 : 2), to afford the product **10** (171 mg) with a yield of 46%. **^{1}H NMR** (200 MHz, $CDCl_3$, δ): 7.47 (s, 4H), 7.45 (s, 4H), 7.36-7.32 (m, 2H), 6.66-6.62 (m, 2H), 3.85 (s, 2H), 0.26 (s, 9H), ppm coincides with the literature.[6]

4-((4-((4-ethynylphenyl)ethynyl)phenyl)ethynyl)aniline (11). A round-bottom flask was charged with **10** (65 mg, 0.166 mmol, 1 eq), dry tetrahydrofuran (THF, 26 mL), and tetra-*n*-

butylammonium fluoride (TBAF in THF 1.0M, 66 mg, 250 μL, 0.25 mmol, 1.5 eq). The reaction mixture was stirred under nitrogen atmosphere at room temperature for 2 h. The mixture was quenched with brine (20 mL), then diluted with CH_2Cl_2 (50 mL), and washed with H_2O (3 x 25 mL). The organic layer was collected and the volatiles removed via rotary evaporation. Without further purification, compound **11** (46 mg) was obtained with a yield of 87%. **^{1}H NMR** (400 MHz, $(CD_3)_2CO$, δ): 7.56-7.51 (m, 8H), 7.28-7.26 (d, 2H), 6.70-6.68 (d, 2H), 5.10 (s, 1H), 3.81 (s, 2H), ppm. **^{13}C NMR** (100 MHz, $(CD_3)_2CO$, δ): 81.24, 83.69, 87.11, 90.91, 91.82, 94.25, 110.62, 114.88, 122.55, 123.32, 124.33, 125.58, 131.99, 132.43, 132.47, 132.98, 133.72, 150.37, ppm.

Synthesis of the chiral donor unit

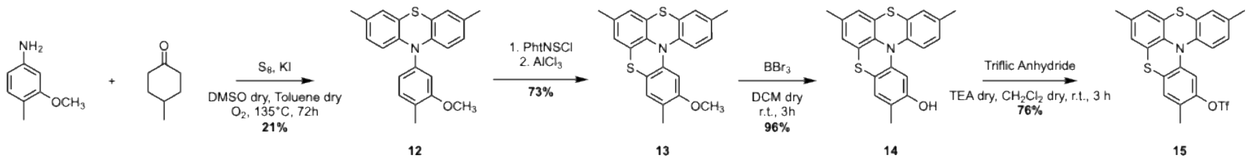

Figure S5. Synthesis of the helicene **15**.

Phenothiazine 12. A Schlenk tube was charged with 3-methoxy-4-methylaniline (2000 mg, 14.6 mmol, 1 eq), S_8 (2803 mg, 87.6 mmol, 6 eq), and KI (484 mg, 2.92 mmol, 0.2 eq). The Schlenk was evacuated and backfilled with N_2 three times and with O_2 three times. Toluene dry (12 mL) and dimethyl sulfoxide dry (DMSO 2.4 mL), and 4-methylcyclohexan-1-one (4906 mg, 5.4 mL, 43.8 mmol, 3 eq) were then added under O_2 atmosphere. The mixture was stirred at 135°C for 72 hours under O_2 atmosphere. The suspension was diluted with CH_2Cl_2 (300 mL) and washed with H_2O (2 x 100 mL) and a solution of $Na_2S_2O_3$ 1M (2 x 100 mL). The organic layer was collected, dried over Na_2SO_4, filtered, and the volatiles were removed via rotary evaporation. The crude was purified by flash chromatography on silica gel with Petroleum Ether : DCM (6 : 1) to obtain **12** (1081 mg) as a yellow solid (21% yield). **^{1}H NMR** (200 MHz, $CDCl_3$, δ): 7.33-7.29 (m, 1H), 6.82-6.79 (m, 4H), 6.65-6.60 (m, 2H), 6.16-6.11 (m, 2H), 3.80 (s, 3H), 2.30 (s, 3H), 2.17 (s, 6H), ppm.

Helicene 13. A round-bottom flask was charged with a solution **12** (480 mg, 1.38 mmol, 1 eq) in DCM dry (7 mL), under N_2 atmosphere. A solution of PhtNSCl (443 mg, 2.1 mmol, 1.5 eq) in DCM dry (15 mL) was added at 0°C. The mixture was stirred at room temperature under N_2 atmosphere for 12 hours. $AlCl_3$ (1.1 eq) was then added and the reaction was stirred for 45 minutes

at room temperature under N_2 atmosphere. The mixture was diluted with 200 mL of DCM and washed with NaOH 1M (2 x 100 mL) and H_2O (2 x 100 mL). The organic layer was collected, dried over Na_2SO_4, filtered, and the volatiles were removed via rotary evaporation. The crude was purified by flash chromatography on silica gel with Petroleum Ether : DCM (3 : 1) to obtain **13** (396 mg) as a yellow solid (73% yield). **^{1}H NMR** (200 MHz, $CDCl_3$, δ): 7.08 (d, J=8.2 Hz, 1H), 7.02 (d, J=1.6 Hz, 1H), 6.92–6.90 (m, 2H), 6.78 (d, J=3.6 Hz, 2H), 6.64 (s, 1H), 3.65 (s, 3H), 2.29 (s, 3H), 2.21 (s, 3H), 2.15 (s, 3H), ppm coincides with the literature.[7]

Helicene 14. A round-bottom flask was charged with a solution **13** (363 mg, 0.96 mmol, 1 eq) in DCM dry (10 mL), under N_2 atmosphere. BBr_3 (721 mg, 2.9 mL, 2.88 mmol, 3 eq) was added at 0°C. The mixture was stirred at room temperature under N_2 atmosphere for 3 hours. The reaction was quenched with ice and diluted with 200 mL of DCM. The mixture was washed with $NaHCO_3$, (2 x 100 mL), NH_4Cl (2 x 100 mL), and H_2O (2 x 100 mL). The organic layer was collected, dried over Na_2SO_4, filtered, and the volatiles were removed via rotary evaporation. The crude was purified by flash chromatography on silica gel with Petroleum Ether : DCM (1 : 5) to obtain **14** (336 mg) as a white solid (96% yield). **^{1}H NMR** (200 MHz, $CDCl_3$, δ): 7.10-7.06 (m, 1H), 6.99-6.89 (m, 3H), 6.77 (bs, 2H), 6.62 (bs, 1H), 4.52 (s, 1H), 2.28 (s, 3H), 2.20 (s, 3H), 2.17 (bs, 3H), ppm coincides with the literature.[8]

Helicene 15. A round-bottom flask was charged with **14** (315 mg, 0.87 mmol, 1 eq), DCM dry (10 mL), and TEA dry (210 mg, 0.29 mL, 2.08 mmol, 2.4 eq), under N_2 atmosphere. Triflic anhydride (318 mg, 0.19 mL, 1.13 mmol, 1.3 eq) was added at 0°C. The mixture was stirred at room temperature under N_2 atmosphere for 3 hours. The reaction was diluted with 200 mL of DCM. The mixture was washed with H_2O (2 x 100 mL), and brine (2x100 mL). The organic layer was collected, dried over Na_2SO_4, filtered, and the volatiles were removed via rotary evaporation. The crude was purified by flash chromatography on silica gel with Petroleum Ether : DCM (4 : 1) to obtain **15** (324 mg) as a white solid (76% yield). **^{1}H NMR** (400 MHz, $CDCl_3$, δ): 7.07–7.02 (m, 3H), 6.98 (bs, 1H), 6.96–6.93 (m, 1H), 6.80 (bs, 1H), 6.76 (bs, 1H), 2.30 (s, 3H), 2.29 (s, 3H), 2.21(s, 3H), ppm.

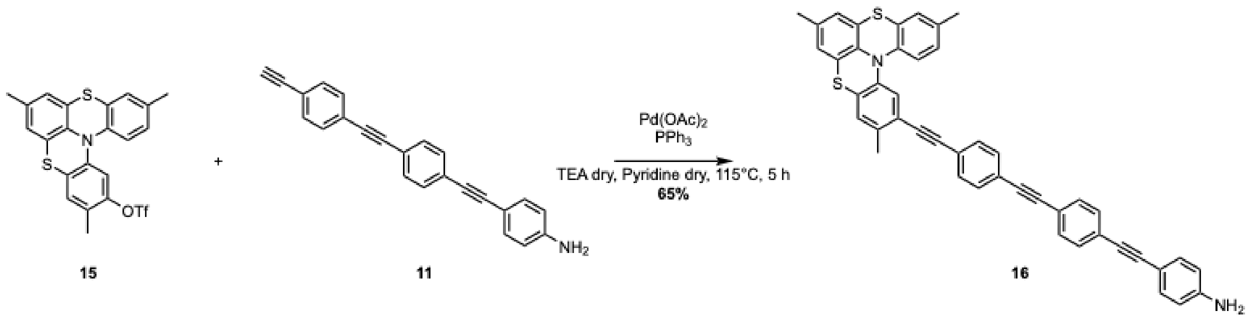

Figure S6. Synthesis of the helicene **16**.

Helicene 16. A Schlenk tube was charged with **15** (100 mg, 0.2 mmol, 1 eq), **11** (115 mg, 0.36 mmol, 1.8 eq), PPh_3 (68 mg, 0.26 mmol, 1.3 eq) and $Pd(OAc)_2$ (2.3 mg, 0.01 mmol, 0.05 eq). Three vacuum-nitrogen cycles were performed, and through syringe dry and degassed triethylamine (728 mg, 1 mL, 7.2 mmol, 36 eq) and pyridine (9.8 g, 10 mL, 124 mmol, 618 eq) were added. The mixture was stirred at 115°C for 5 hours. The suspension was diluted with CH_2Cl_2 (50 mL) and washed with H_2O (2 x 50 mL). The organic layer was collected, dried over Na_2SO_4, filtered, and the volatiles were removed via rotary evaporation. The crude was purified by flash chromatography on silica gel with CH_2Cl_2 to obtain **16** (86 mg) as a yellow solid (65% yield). **^{1}H NMR** (400 MHz, $CDCl_3$, *δ*): 7.48-7.45 (m, 8H), 7.35-7.33 (d, 2H), 7.28 (s, 1H), 7.11-7.09 (m, 1H), 7.05-7.02 (m, 2H), 6.97-6.94 (m, 1H), 6.80-6.78 (m, 2H), 6.64-6.62 (d, 2H), 3.83 (s, 2H), 2.44 (s, 3H), 2.30 (s, 3H), 2.21 (s, 3H), ppm. **^{13}C NMR** (100 MHz, $CDCl_3$, *δ*): 147.00, 140.57, 140.03, 137.07, 136.28, 134.71, 134.46, 133.17, 131.64, 131.61, 131.51, 131.39, 128.56, 128.47, 128.30, 127.96, 126.84, 126.31, 126.09, 125.68, 123.27, 123.08 123.05, 121.94, 120.33, 114.86, 112.40, 93.78, 92.53, 91.37, 90.80, 89.93, 87.32, 20.77, 20.51, 20.08, ppm. **Melting Point**: 255°C. **IR** (ATR neat) ν = 3474, 3378, 3035, 2917, 2856, 2205, 1616, 1519, 1449, 1279, 827 cm^{-1}. **Elem. Anal.** calcd for $C_{45}H_{30}N_2S_2$: C, 81.54; H, 4.56; N, 4.23; S, 9.67. Found: C, 81.46; H, 4.51; N, 4.18; S, 9.55. **ESI-MS**: 663.34 $(M+H)^+$.

Synthesis of the chiral donor-acceptor dyad

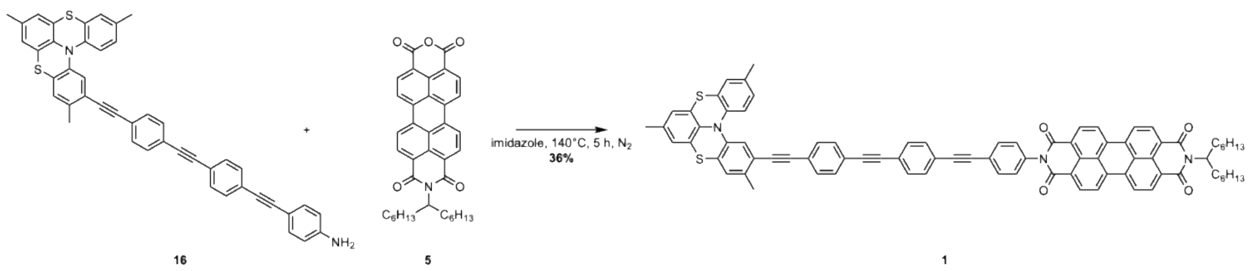

Figure S7. Synthesis of the chiral dyad **1**.

Chiral Dyad 1. A round-bottom flask was charged with **5** (40 mg, 0.07 mmol, 1 eq), **13** (56 mg, 0.084 mmol, 1.2 eq), and imidazole (68 mg, 3.36 mmol, 48 eq). The reaction mixture was stirred at 140°C for 5 h, under nitrogen flux. Once cooled to room temperature, NH_4Cl (15 mL) was added to the mixture, and left stirring for 1 h. The mixture was then diluted with CH_2Cl_2 (50 mL) and washed with H_2O (2 x 25 mL). The organic layer was collected, dried over Na_2SO_4, filtered, and the volatiles were removed via rotary evaporation. The crude was purified by flash chromatography on silica gel with Petroleum Ether : CH_2Cl_2 (1 : 7) to obtain **1** (31 mg) as a red solid (36% yield). **^{1}H NMR** (400 MHz, $CDCl_3$, δ): 8.63-8.47 (m, 8H), 7.73-7.71 (m, 2H), 7.51 (s, 4H), 7.46-7.43 (m, 4H), 7.35-7.33 (m, 2H), 7.15 (s, 1H), 7.04-7.00 (m, 2H), 6.93-6.91 (m, 2H), 6.77-6.72 (m, 2H), 5.19 (m, 1H), 2.37 (s, 3H), 2.29-2.21 (m, 8H), 1.91 (m, 2H), 1.26 (m, 16H), 0.84 (t, J = 8 Hz, 6H), ppm. **^{13}C NMR** (100 MHz, $CDCl_3$, δ): 163.40, 140.39, 140.02, 136.96, 136.18, 135.13, 134.76, 134.49, 134.11, 132.72, 131.82, 131.71, 131.53, 129.76, 129.48, 129.03, 128.47, 128.38, 128.33, 127.83, 126.83, 126.57, 126.32, 126.29, 126.11, 125.65, 125.07, 123.88, 123.32, 123.23, 123.14, 123.12, 123.07, 122.99, 121.84, 120.26, 93.72, 91.20, 90.91, 89.88, 55.01, 32.53, 31.93, 29.38, 27.17, 22.75, 20.77, 20.53, 20.05, 14.20, ppm. **Melting Point**: 223°C. **IR** (ATR solid) ν = 3037, 2921, 2853, 2213, 1914, 1696, 1656, 1340, 809 cm^{-1}. **Elem. Anal.** calcd for $C_{82}H_{63}N_3O_4S_2$: C, 80.83; H, 5.21; N, 3.45; O, 5.25; S, 5.26. Found: C, 80.80; H, 5.13; N, 3.38; O, 5.65; S, 5.04. **ESI-MS**: 1217.43 $(M)^{\bullet -}$.

Synthesis of the achiral donor unit

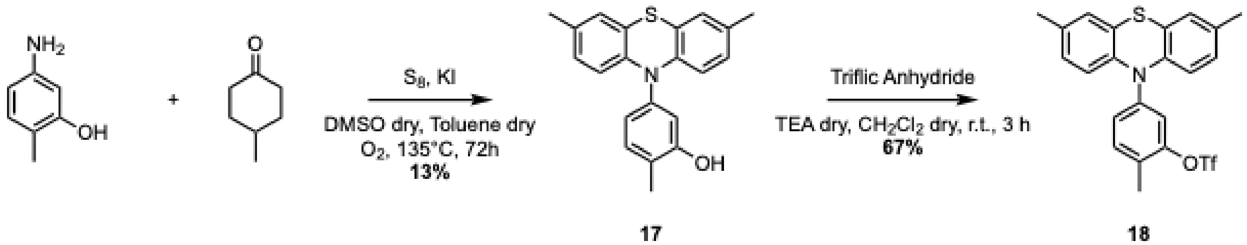

17

18

Figure S8. Synthesis of the phenothiazine **18**.

Phenothiazine 17. A Schlenk tube was charged with 2-methyl-5-aminophenol (500 mg, 4.1 mmol, 1 eq), S_8 (780 mg, 24.4 mmol, 6 eq), and KI (135 mg, 0.813 mmol, 0.2 eq). The Schlenk was evacuated and backfilled with N_2 three times and with O_2 three times. Toluene dry (3.3 mL) and dimethyl sulfoxide dry (DMSO 0.7 mL), and 4-methylcyclohexan-1-one (1.4 g, 1.5 mL, 12.2

mmol, 3 eq) were then added under O_2 atmosphere. The mixture was stirred at 135°C for 72 hours under O_2 atmosphere. The suspension was diluted with CH_2Cl_2 (150 mL) and washed with H_2O (2 x 100 mL) and a solution of $Na_2S_2O_3$ 1M (2 x 100 mL). The organic layer was collected, dried over Na_2SO_4, filtered, and the volatiles were removed via rotary evaporation. The crude was purified by flash chromatography on silica gel with Petroleum Ether : Ethyl Acetate (8 : 1) to obtain **17** (182 mg) as a yellow solid (13% yield). **^{1}H NMR** (400 MHz, $CDCl_3$, δ):7.29-7.27 (d, J = 8 Hz, 1H), 6.83-6.82 (m, 3H), 6.73 (s, 1H), 6.65-6.63 (m, 2H), 6.21-6.19 (d, J = 8 Hz, 2H), 4.92 (s, 1H), 2.32 (s, 3H), 2.18 (s, 6H), ppm. **^{13}C NMR** (100 MHz, $CDCl_3$, δ): 155.53, 141.97, 140.56, 132.69, 131.93, 127.39, 127.25, 123.60, 122.25, 120.52, 116.41, 116.31, 20.31, 15.72, ppm.

Phenothiazine 18. A round bottom flask was charged with phenothiazine **17** (150 mg, 0.44 mmol, 1 eq), TEA dry (91 mg, 0.125 mL, 0.90 mmol, 2 eq) and CH_2Cl_2 dry (5 mL). Triflic anhydride (152 mg, 91 µL, 0.54 mmol, 1.2 eq) was added dropwise at 0°C under stirring. The reaction mixture was stirred under N_2 atmosphere for 4h. The mixture was diluted with CH_2Cl_2 (100 mL) and washed with brine (2 x 100 mL) and H_2O (2 x 100 mL). The organic layer was collected, dried over Na_2SO_4, filtered, and the volatiles were removed via rotary evaporation. The crude was purified by flash chromatography on silica gel with Petroleum Ether : Ethyl Acetate (20 : 1) to obtain **18** (138 mg) as a pale yellow solid (68% yield). **^{1}H NMR** (400 MHz, $CDCl_3$, δ): 7.37-7.35 (d, J = 8 Hz, 1H), 7.18-7.14 (m, 2H), 7.01-6.99 (m, 2H), 6.84-6.81 (m, 2H), 6.48-6.46 (m, 2H), 2.41 (s, 3H), 2.25 (s, 6H), ppm. **^{13}C NMR** (100 MHz, $CDCl_3$, δ): 149.23, 142.77, 140.83, 133.71, 133.45, 128.09, 127.73, 125.62, 125.04, 119.45, 118.99, 20.54, 16.17, ppm.

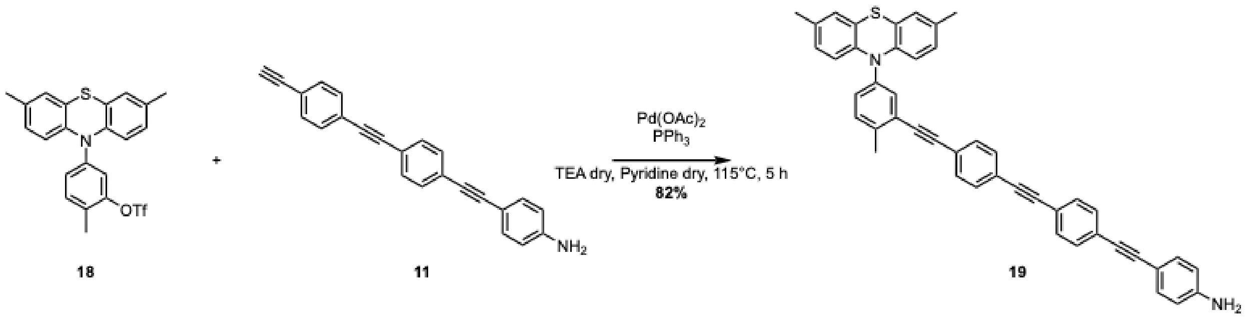

Figure S9. Synthesis of the phenothiazine **19**.

Phenothiazine 19. A Schlenk tube was charged with **18** (80 mg, 0.173 mmol, 1 eq), **11** (99 mg, 0.311 mmol, 1.8 eq), PPh_3 (60 mg, 0.225 mmol, 1.3 eq) and $Pd(OAc)_2$ (2 mg, 0.009 mmol, 0.05 eq). Three vacuum-nitrogen cycles were performed, and through syringe dry and degassed

triethylamine (729 mg, 1 mL, 7.2 mmol, 42 eq) and pyridine (9.8 g, 10 mL, 124 mmol, 716 eq) were added. The mixture was stirred at 115°C for 5 hours. The suspension was diluted with CH_2Cl_2 (100 mL) and washed with H_2O (2 x 50 mL). The organic layer was collected, dried over Na_2SO_4, filtered, and the volatiles were removed via rotary evaporation. The crude was purified by flash chromatography on silica gel with CH_2Cl_2 : Petroleum Ether = 4 : 1, to obtain **19** (90 mg) as a yellow solid (82% yield). **^{1}H NMR** (200 MHz, $CDCl_3$, δ): 7.51-7.48 (m, 12H), 7.37-7.33 (m, 2H), 6.86-6.85 (m, 1H), 6.67-6.72 (m, 4H), 6.19-6.15 (m, 2H), 3.85 (s, 2H), 2.60 (s, 3H), 2.18 (s, 6H) ppm.

Synthesis of the achiral donor-acceptor dyad

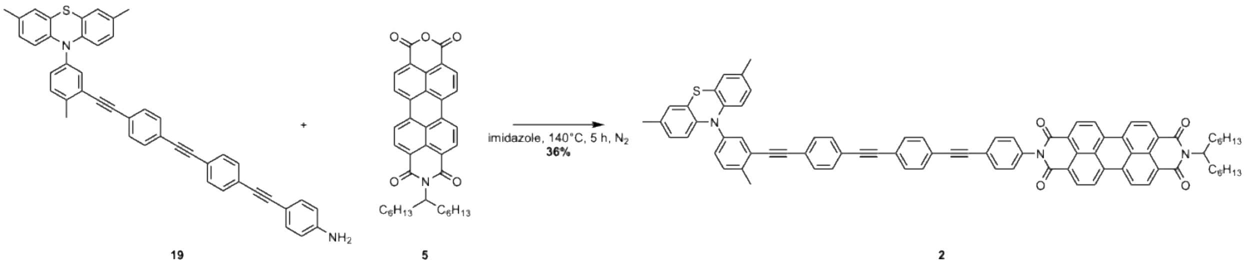

Figure S10. Synthesis of the achiral dyad **2**.

Achiral Dyad 2. A round-bottom flask was charged with **5** (38 mg, 0.066 mmol, 1 eq), **19** (50 mg, 0.079 mmol, 1.2 eq), and imidazole (216 mg, 3.17 mmol, 48 eq). The reaction mixture was stirred at 140°C for 5 h, under nitrogen flux. Once cooled to room temperature, NH_4Cl (14 mL) was added to the mixture, and left stirring for 1 h. The mixture was then diluted with CH_2Cl_2 (50 mL) and washed with H_2O (2 x 50 mL). The organic layer was collected, dried over Na_2SO_4, filtered, and the volatiles were removed via rotary evaporation. The crude was purified by flash chromatography on silica gel with Petroleum Ether : CH_2Cl_2 (1 : 3) to obtain **2** (16 mg) as a red solid (20% yield). **^{1}H NMR** (400 MHz, $CDCl_3$, δ): 8.71-8.57 (m, 8H), 7.74-7.72 (m, 2H), 7.54-7.51 (m, 8H), 7.43-7.41 (m, 1H), 7.39-7.37 (m, 2H), 7.24-7.22 (m, 1H), 6.83 (s, 1H), 6.67-6.65 (m, 2H), 6.18-6.16 (m, 2H), 5.19 (m, 1H), 2.60 (s, 2H), 2.27-2.18 (m, 8H), 1.89 (m, 2H), 1.39-1.24 (m, 19H), 0.84 (t, J = 8 Hz, 6H), ppm. **^{13}C NMR** (100 MHz, $CDCl_3$, δ): 163.52, 141.89, 139.90, 139.44, 135.31, 135.18, 133.61, 132.77, 132.06, 131.96, 131.94, 131.83, 131.77, 131.73, 131.71, 131.63, 129.61, 129.03, 127.44, 127.34, 126.75, 126.47, 123.47, 123.31, 123.27, 123.17,

120.73, 116.44, 94.39, 91.17, 90.90, 90.18, 89.63, 55.01, 32.52, 31.91, 29.37, 27.12, 22.74, 20.66, 20.33, 14.20, ppm. **ESI-MS**: 1187.20 $(M)^{\bullet -}$.

2. HPLC and Circular Dichroism Spectroscopy

Experimental HPLC Analytical (250 × 4.6 mm/ 5 μm) column packed with CHIRALPAK® IA chiral stationary phase was purchased from Chiral Technologies Europe. The HPLC resolution of products was performed with HPLC Semipreparative (250 x 10 mm/ 5 μm) column packed with CHIRALPAK® IG chiral stationary phase, purchased from Chiral Technologies Europe, on a HPLC waters Alliance 2695 equipped with a 200 μL loop injector and a spectrophotometer UV waters PDA 2996, The mobile phase, delivered at a flow rate of 1.5 mL/min, was hexane : CH_2Cl_2 : IPA = 50 : 50 : 4 for the chiral dyad 1.

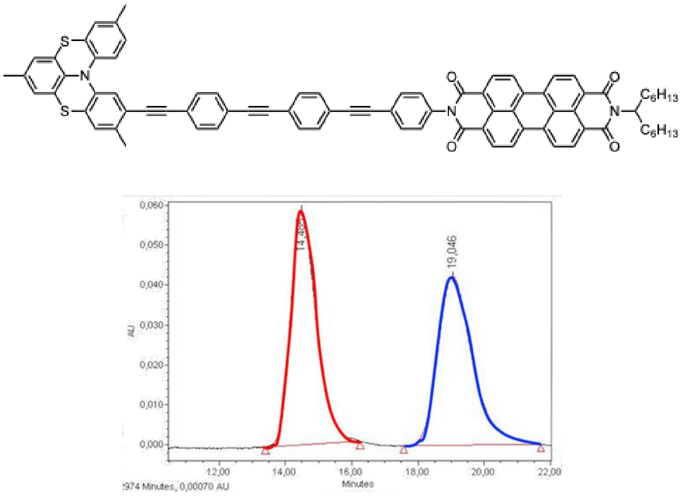

Figure S11. HPLC chromatogram of the racemic mixture of the **chiral dyad 1**.

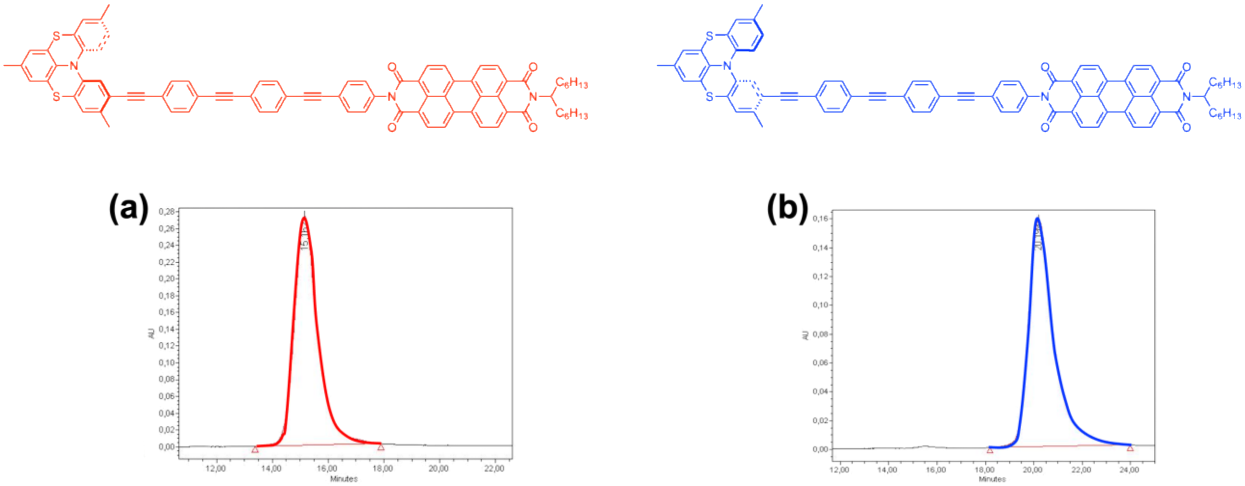

Figure S12. (a) HPLC chromatogram of (*P*)-**1**. (b) HPLC chromatogram of (*M*)-**1**.

Circular Dichroism (CD) spectra were recorded using the J-1500 Circular Dichroism Spectrophotometer, in CH_2Cl_2 ($2.4x10^{-5}$M).

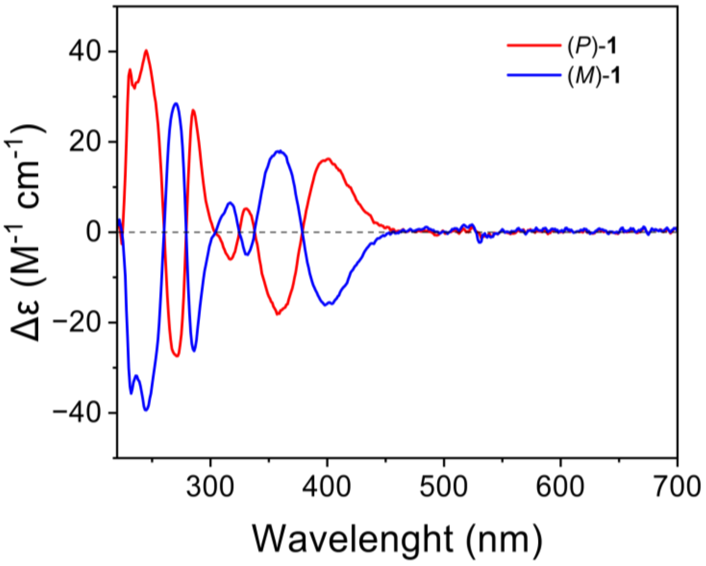

Figure S13. CD spectra of (*P*)-**1** and (*M*)-**1**.

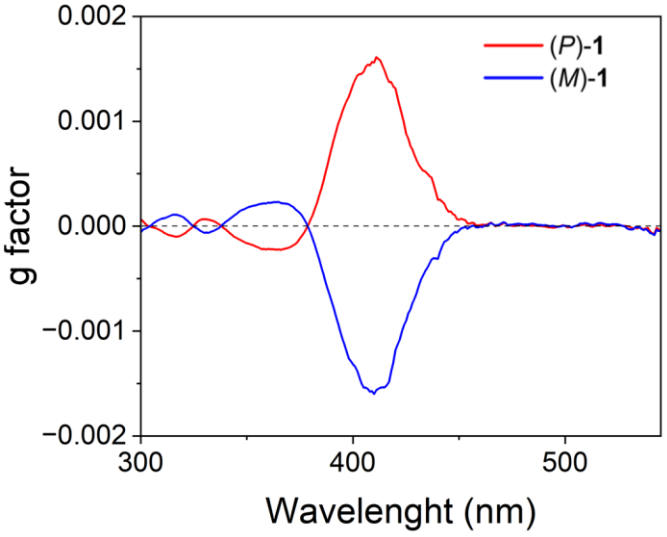

Figure S14. Dissymmetry factor $g_{dis}=2(\varepsilon_L-\varepsilon_R)/(\varepsilon_L+\varepsilon_R)$ of (*P*)-**1** and (*M*)-**1**.

3. Cyclic Voltammetry

Electrochemical measurements were performed on a CH Instrument Model 622 electrochemical working station. Measurements were made using a 1.0 mm diameter glassy carbon working electrode, a platinum wire auxiliary electrode, and a silver wire reference electrode in 0.1 M solutions of n-butylammonium hexafluorophosphate ($TBAPF_6$), as supporting electrolyte, in CH_2Cl_2 purged with argon, at a potential scan rate of 100 mV s^{-1}. Potentials are referenced to the ferrocene/ferrocinium, Fc/Fc+ redox couple. Before each use the cell was dried in the oven, the glassy carbon electrode was polished, the platinum wire auxiliary electrode was washed with acetone and dried, the silver wire reference electrode was polished with sandpaper and washed with H_2O and acetone and dried. Argon was continuously flushed through during the measurements.

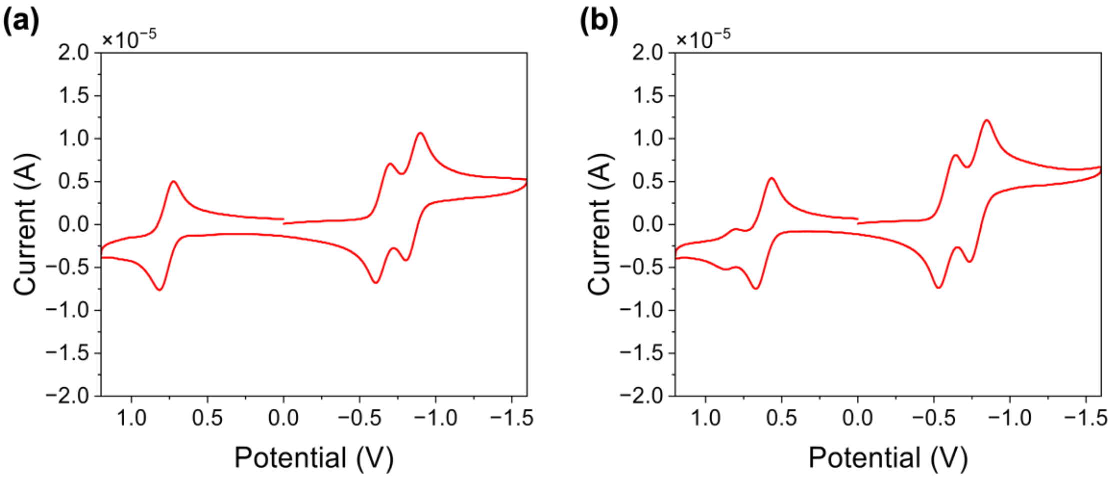

Figure S15. **(a)** Cyclic Voltammetry recorded in CH_2Cl_2 of the **rac1**, redox potentials +0.84 V, -0.58 V, -0.77 V. **(b)** Cyclic Voltammetry recorded in CH_2Cl_2 of the achiral dyad **2**, redox potentials +0.8 V, +0.62 V, -0.59 V, -0.79 V.

Weller Equation Analysis. The Weller equation[9] was applied to these data to correct for the different dielectric constants between CH_2Cl_2, the solvent electrochemical potentials were measured in, and 2-Me-THF and toluene, the solvents transient experiments were performed in, as follows:

$$\Delta G = E_{ox} - E_{red} - \left(\frac{e^2}{r_{DA}\varepsilon_S}\right) + \left(e^2\left(\frac{1}{2r_D} + \frac{1}{2r_A}\right)\left(\frac{1}{\varepsilon_S} - \frac{1}{\varepsilon_{SP}}\right)\right)$$

Eqn. S1

Where E_{ox} and E_{red} are the oxidation and reduction potentials of the donor and acceptor, respectively, in the solvent electrochemical measurements were taken with a static dielectric constant ε_{SP}, e is the charge of an electron, r_{DA} is the ion pair distance, r_D and r_A are the ionic radii of donor and acceptor (both calculated from the structures given by DFT calculations), and ε_S is the static dielectric constant of the solvent we are comparing against.

4. Transient absorption spectroscopy

Experimental

Femtosecond and nanosecond transient absorption (TA) measurements were performed in 2-MeTHF at 85 K using a commercial Ti:sapphire laser system (Tsunami oscillator/Spitfire amplifier, Spectra-Physics), as described previously.[10,11] Pump pulses at 530 nm were generated with a collinear optical parametric amplifier (TOPAS-Prime, Light Conversion, LLC) and attenuated to < 1 μJ/pulse. To suppress polarization-dependent dynamics, the pump pulses were depolarized using a DPU-25-A (Thorlabs Inc.). Samples (optical density 0.4-0.6 at 530 nm in a 2 mm cuvette) were mounted in a VNF-100 cryostat (Janis Research Company, LLC) between two copper plates to ensure good thermal contact. The temperature was monitored and controlled with a Cryo-Con 32B controller (Cryogenic Control Systems, Inc.).

TA spectra were corrected for group delay dispersion and scattered light using Surface Xplorer (Ultrafast Systems, LLC) prior to kinetic analysis. Global kinetic analysis was then performed to extract evolution-associated spectra and kinetic parameters, following procedures described previously.[12] The samples were observed to show some degradation during the course of the fsTA experiment, though the observed degradation only decreased the signal amplitude over time and did not distort the spectral shape. Since the time delays in the fsTA measurement were sampled monotonically this degradation is imprinted into the raw kinetic data; the nsTA time delays are sampled randomly and thus do not suffer this issue. To correct for the sample degradation, a second dataset was acquired wherein only one time delay was sampled for the same total number of steps and exposure time as for the original dataset.[13] The normalized, wavelength-averaged TA signal as a function of acquisition time was fit to a biexponential decay. The original data were then divided by this fit to remove the effects of degradation, and then merged and analyzed as described in the text.

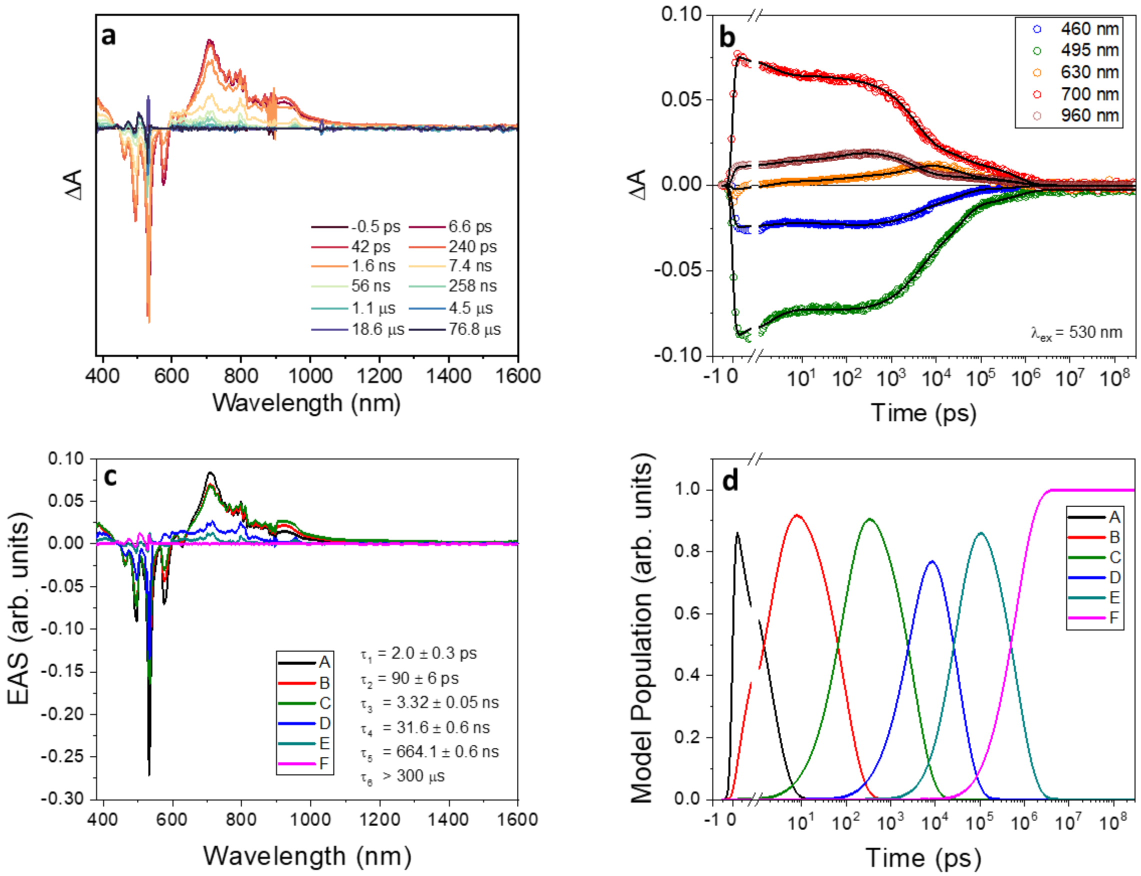

Figure S16. (a) Transient Absorption (TA) spectra of dyad **rac1** in 2-MeTHF at 85K, excited at 530 nm and recorded at the indicated pump-probe delay times. (b) Kinetic traces at representative wavelengths with corresponding global fits. (c) Evolution-associated spectra (EAS) obtained by the global fit analysis. (d) Population dynamics based on a sequential kinetic model A → B → C → D → E → F. States A, B, and C correspond to the singlet excited state of PDI; this state relaxes and undergoes charge transfer with multiple time constants due to distributed kinetics in the low-temperature matrix. States D and E are assigned to the charge-transfer state between the PDI radical anion and the dithia-aza[4]helicene radical cation, which relaxes and recombines with several time constants, also reflecting distributed kinetics. State F is attributed to the PDI triplet state.

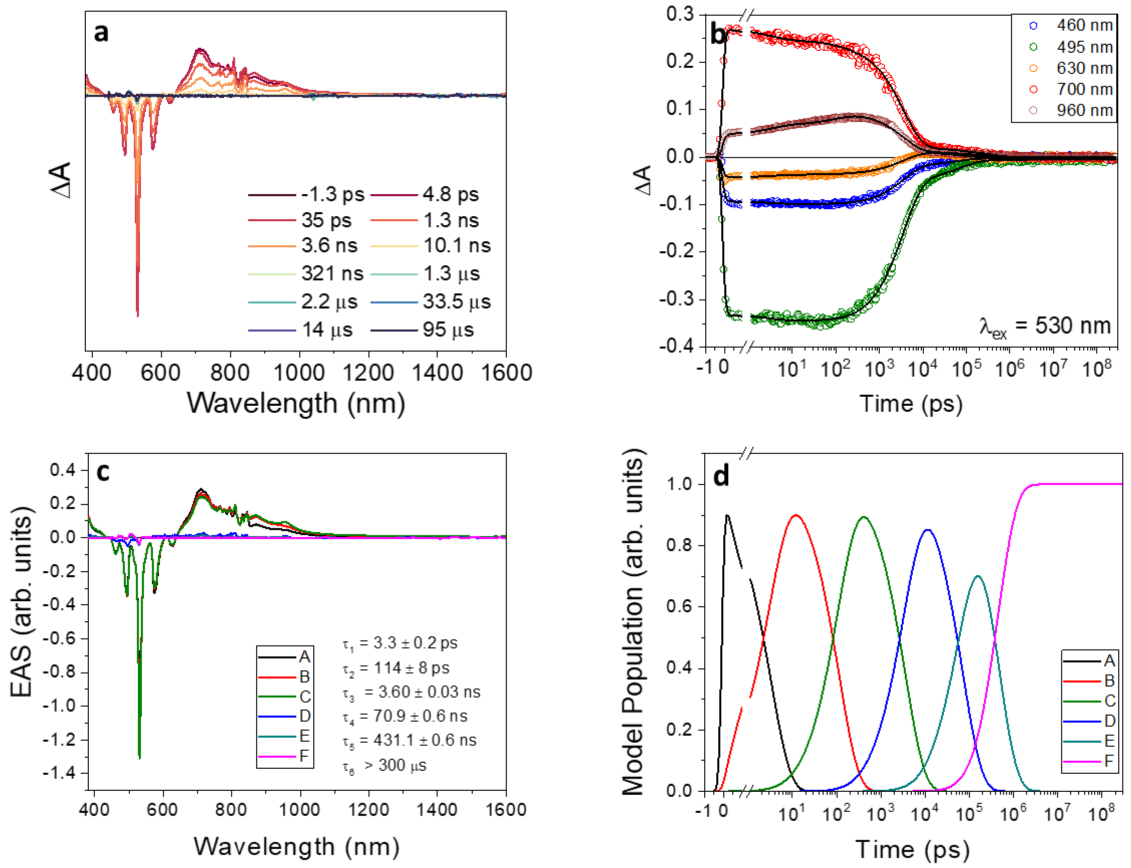

Figure S17. (a) Transient Absorption (TA) spectra of the achiral dyad **2** in 2-MeTHF at 85K, excited at 530 nm and recorded at the indicated pump-probe delay times. (b) Kinetic traces at representative wavelengths with corresponding global fits. (c) Evolution-associated spectra (EAS) obtained by the global fit analysis. (d) Population dynamics based on a sequential kinetic model A → B → C → D → E → F. States A, B, and C correspond to the singlet excited state of PDI; this state relaxes and undergoes charge transfer with multiple time constants due to distributed kinetics in the low-temperature matrix. States D and E are assigned to the charge-transfer state between the PDI radical anion and the phenothiazine radical cation, which relaxes and recombines with several time constants, also reflecting distributed kinetics. State F is attributed to the PDI triplet state.

5. Computational details

UV–Vis absorption spectra were computed using TD-DFT based on ground-state geometries optimized via DFT. The optimal agreement with experimental data was achieved by employing the CAM-B3LYP functional for geometry optimization in conjunction with the 6-31G(d) basis set. Subsequent TD-DFT calculations were performed using the B3LYP functional, considering 100 excited states. All TD-DFT computations were carried out without invoking the Tamm–Dancoff approximation, and the RIJCOSX approximation was applied to accelerate the evaluation of excitation energies and analytical gradients.

The selection of B3LYP for excited-state calculations was justified by the observed spatial misalignment between the thia-bridged [4]helicene moiety and the perylene diimide (PDI) core, which persisted regardless of the bridging ligand length. Although B3LYP is known to underestimate charge-transfer (CT) states, the geometric localization of donor and acceptor orbitals in this system led to improved spectral agreement compared to the long-range corrected CAM-B3LYP functional, which typically offers superior CT state descriptions.

To correct for systematic deviations in computed transition energies, the theoretical spectra were uniformly shifted to align with experimental absorption maxima at ca 550 nm. Dispersion interactions were accounted for using the DFT-D3(BJ) method with Becke–Johnson damping. Solvent effects were modelled using the conductor-like screening model (COSMO), with the dielectric constant set to that of toluene ($\varepsilon = 2.40$).

UV-vis and CD spectra are reported in Figure S16.

The computational protocol illustrated above has been applied to the **(P)-1** and **2** systems.

The *g* and hyperfine values for **1** and **2** were calculated extrapolating the dithia-aza[4]helicene and N-arylphenothiazine from the complete dyad saturating the broken carbon-carbon bond between them and the bridge with a hydrogen atom. For the former, we have removed one electron to render it paramagnetic, thereby emulating the CT states. The geometries were allowed to relax for both species. All calculations were performed within the B3LYP/6-31G*(Toluene) framework and including spin-orbit coupling. The computed values are reported in Table 1.

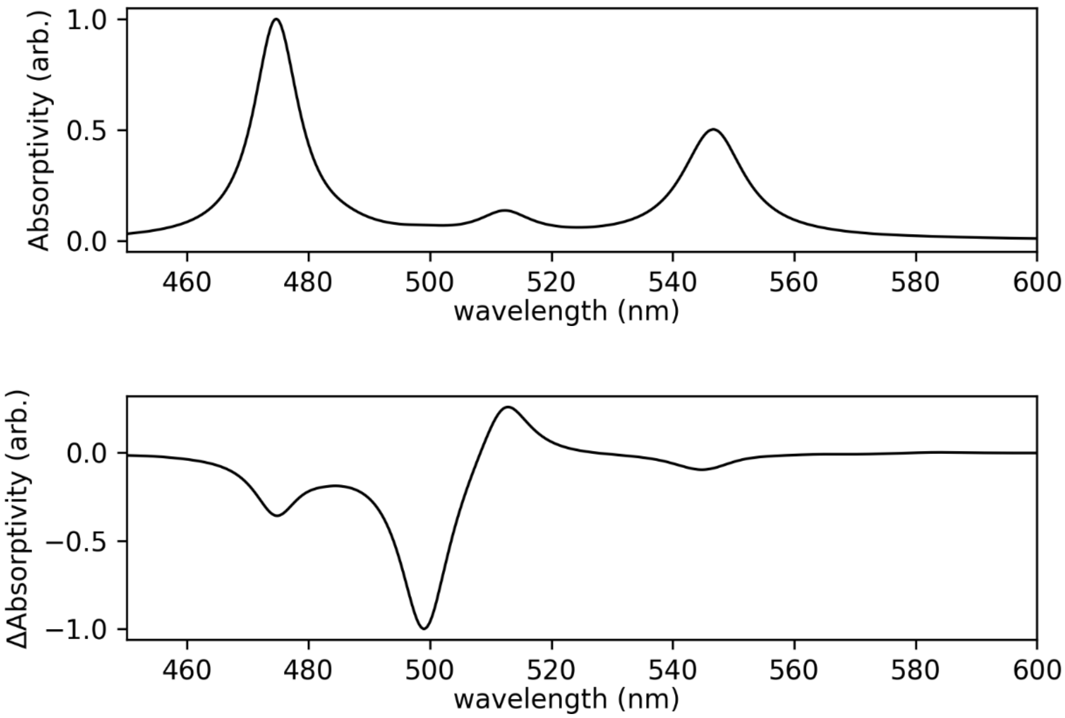

Figure S18. The UV-vis and ECD spectra computed for P by DFT (see computational details). The spectrum has been blue-shifted by 140 nm to align it to experimental one.

The computed UV–vis and ECD spectra reproduce qualitatively the main experimental features, despite the expected TD-DFT red-shift and the absence of vibronic structure in the calculations. The relative intensities and positions of the two principal absorption bands are consistent with experiment, and the sign pattern of the Cotton effects is correctly captured, with a dominant negative feature followed by a positive one.

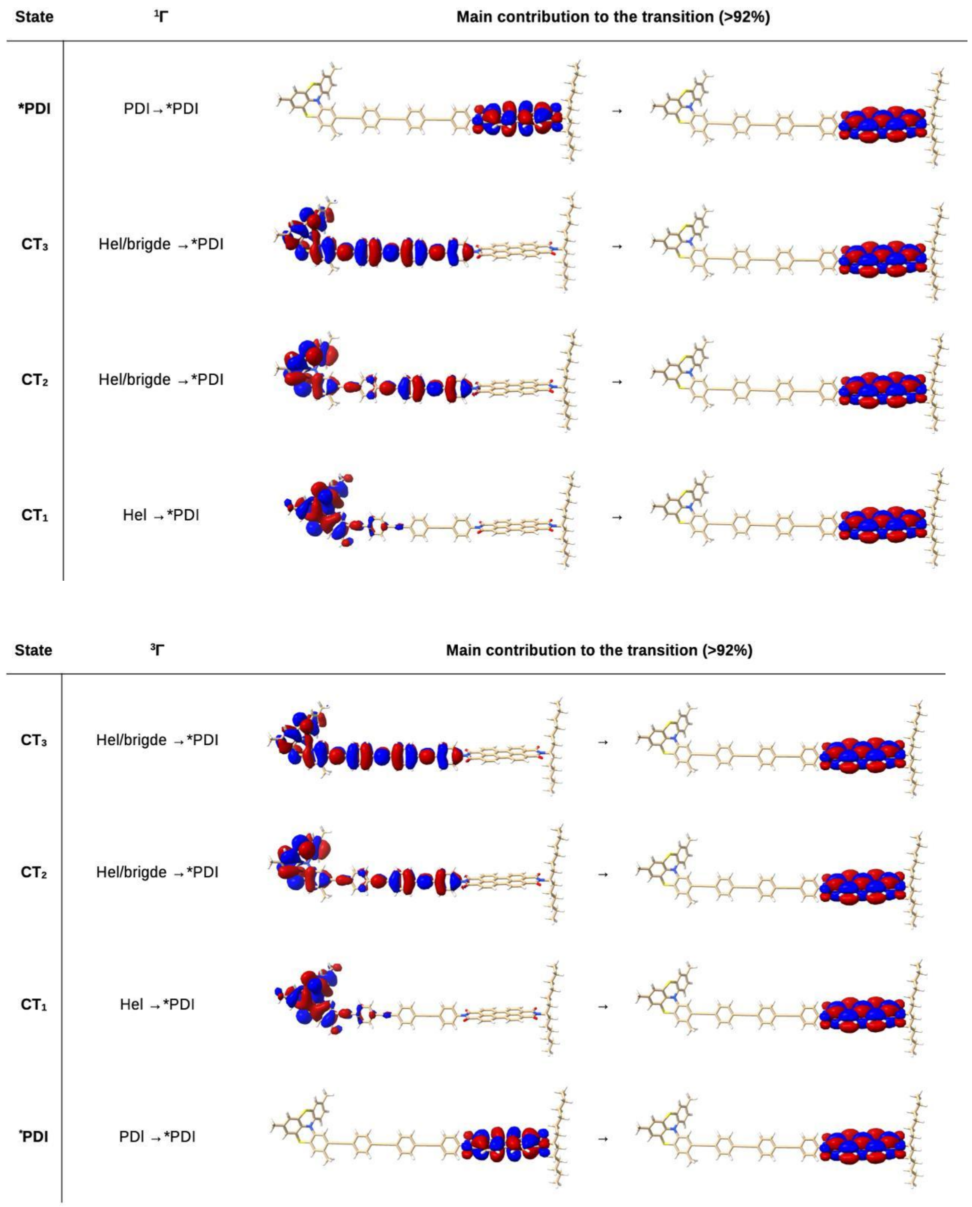

Figure S19. Computed molecular orbitals involved in the CT_{1-3} and *PDI excitations for $^{1-3}\Gamma$ states.

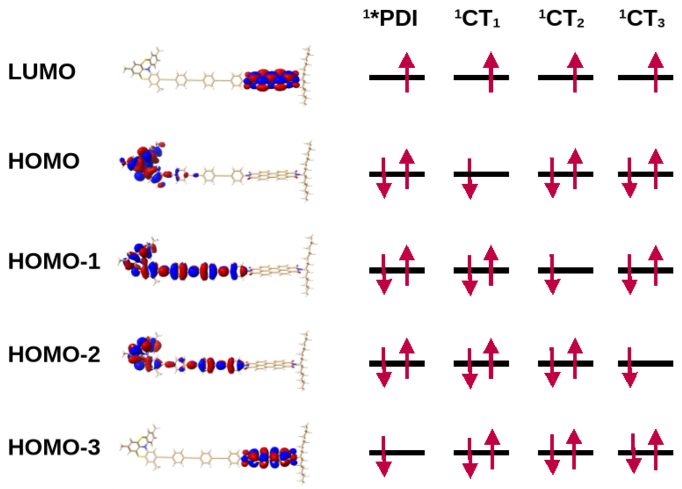

Figure S20. Calculated frontier molecular orbitals and electronic configurations of the PDI-dithiaaza[4]helicene dyad. Left: Spatial distribution of the LUMO and HOMO to HOMO-3 orbitals calculated at the TD-DFT level. The LUMO and HOMO-3 are strictly localized on the perylene diimide (PDI) acceptor, while the HOMO is localized on the dithiaaza[4]helicene donor. HOMO-1 and HOMO-2 show significant delocalization across the bridge. Right: Schematic representation of the electronic configurations for the locally excited singlet state of PDI (^{1*}PDI) and the three primary charge-transfer states ($^1CT_1$, $^1CT_2$, and $^1CT_3$). The ^{1*}PDI state arises from the (HOMO-3) to LUMO transition, whereas the CT states result from the reorganization of the electron density; this leads to electronic configurations where the unpaired electrons reside in the PDI-localized LUMO and the respective helicene-centered HOMO (CT_1), HOMO-1 (CT_2), and HOMO-2 (CT3).

6. Electron Paramagnetic Resonance

Experimental

X-band EPR measurements were performed on a Bruker Elexsys E580 spectrometer equipped with a split-ring resonator (Bruker ER4118X-MS3). Sample temperature was controlled using an Oxford Instruments CF935 continuous-flow cryostat cooled with liquid nitrogen and an ITC503S temperature controller.

For X-band Time-Resolved Continuous-Wave EPR (TREPR), the sample was photoexcited at 530 nm using 7 ns pulses from an optical parametric oscillator (Spectra-Physics BasiScan) pumped by the 355 nm output of a frequency-tripled Nd:YAG laser (Spectra-Physics Quanta-Ray Lab-150-10H) operating at 10 Hz. The unpolarized laser light was delivered to the resonator via an optical fiber and a collimator positioned outside the cryostat window, yielding approximately 1 mJ per pulse with a beam diameter (FWHM) of ~5 mm. Following photoexcitation, transient magnetization time traces were recorded as a function of magnetic field using direct diode detection under continuous microwave irradiation. Data processing involved subtracting the background signal prior to the laser pulse from each kinetic trace (at a given magnetic field), followed by subtracting the off-resonance signal from each spectrum (at a given time).

For X-band echo-detected EPR (EDEPR), spectra were recorded using the standard two-pulse Hahn echo sequence ($\pi/2 - \tau - \pi$), with pulse lengths of $\pi/2$ = 16 ns and π = 32 ns.

W-band EPR measurements were carried out on a Bruker Elexsys E680 spectrometer equipped with a cylindrical resonator (Bruker EN-680-1021H), with temperature control provided by the same CF935 continuous-flow cryostat and ITC503S controller. At W-band, pulses were shaped using an arbitrary waveform generator (AWG). Pulse lengths of $\pi/2$ = 20 ns and π = 40 ns were used.

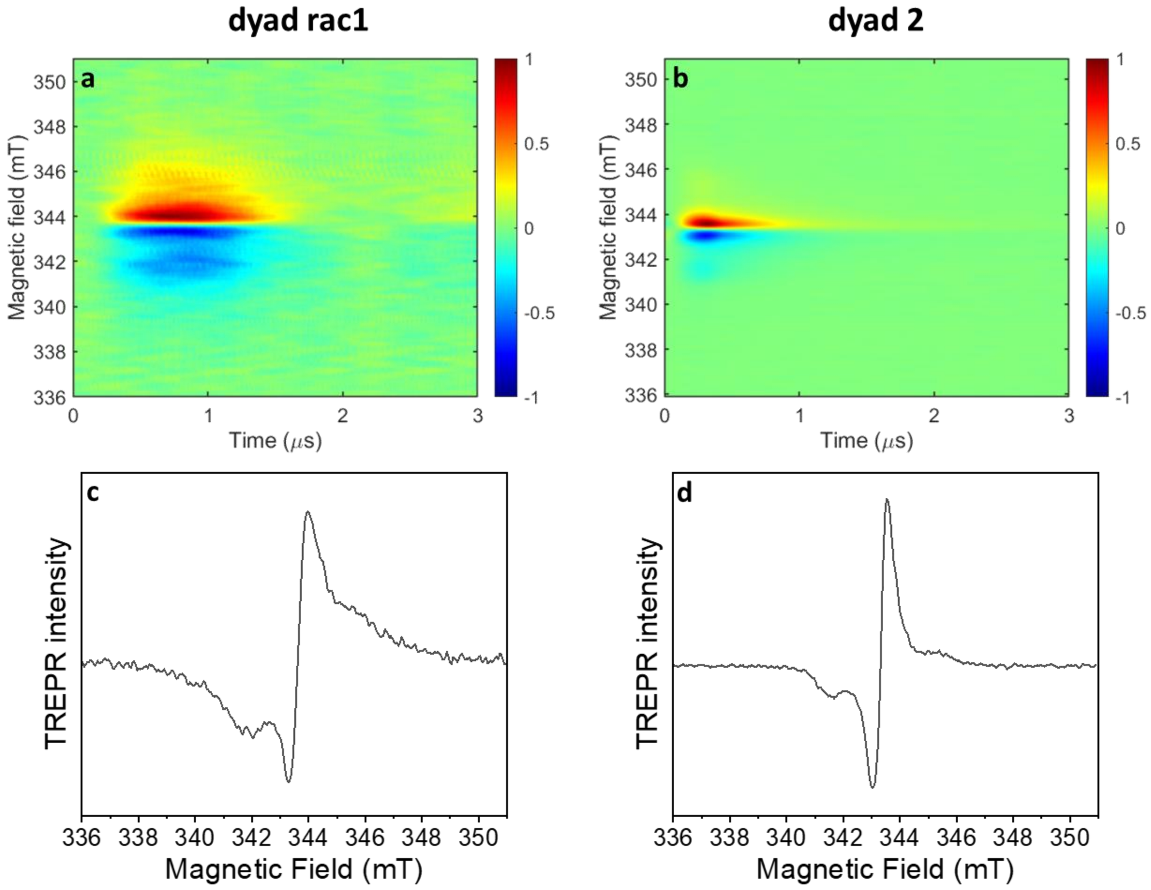

Figure S21. (a,b) Normalized 2D experimental TREPR contour plots of dyads **rac1** and **2** in frozen toluene solution at 85 K following a 530 nm laser pulse (7 ns, 2 mJ). Color scale: red = enhanced absorption, blue = emission, green = baseline. (c,d) Normalized 1D experimental TREPR spectra of chiral and achiral dyads, recorded 500 ns after the laser pulse (integration window = 200 ns).

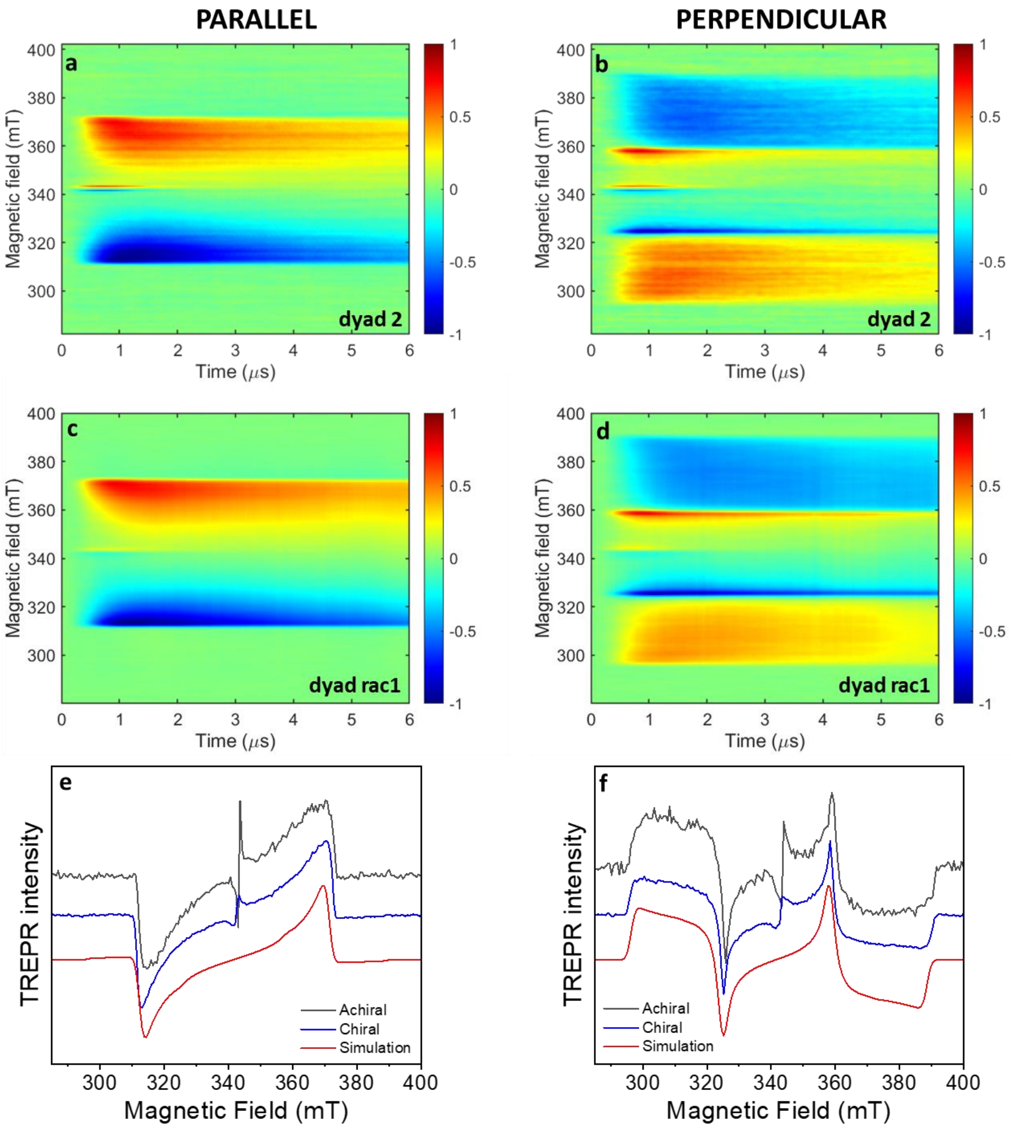

Figure S22. (a-d) Normalized 2D experimental TREPR contour plots of dyads **racl** and **2** aligned in the nematic liquid crystal 5CB at 85 K following a 530 nm laser pulse (7 ns, 2 mJ). Spectra were measured with the liquid crystals oriented parallel (left column) and perpendicular (right column) to the magnetic field. Color scale: red = enhanced absorption, blue = emission, green = baseline. (e,f) Normalized 1D experimental TREPR spectra of achiral (black) and chiral (blue) dyads, along with spectral simulations (red), recorded 1 μs after the laser pulse (integration window = 200 ns). The dyads are oriented with their long axis parallel (e) and perpendicular (f) to the external magnetic field. The broad spectral features spanning 290 to 390 mT are attributed to the PDI excited triplet state and were simulated using the parameters listed in Table S1.

	Dyad **rac1**	Dyad **2**
**[D, E] (MHz)**	[1300, -120]	[1300, -120]
**[p_{-1}, p_0, p_{+1}]**	[0, 1, 0]	[0, 1, 0]
**LW (mT)**	2.5	2.5
**Ordering**	-3	+2.5

Table S1. Zero-field splitting parameters (D and E in MHz), triplet sublevel populations (p_{-1}, p_0, p_{+1}), Gaussian linewidths (LW in mT), and ordering parameters used in the spectral simulations of the triplet spectra shown in Figure S20. The simulations reproduce the characteristic triplet spin polarization pattern expected from a radical pair intersystem crossing mechanism.

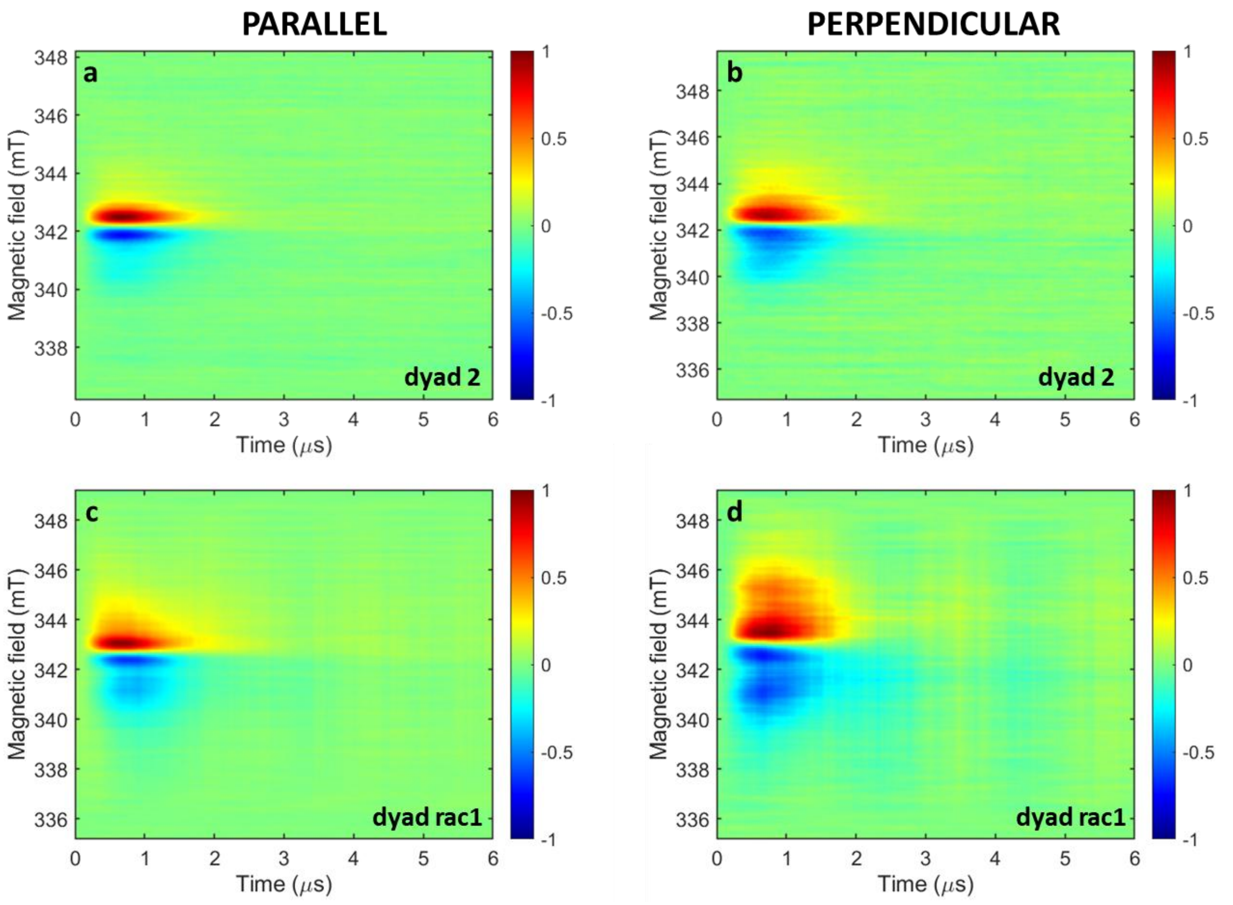

Figure S23. Normalized 2D experimental TREPR contour plots of dyads **rac1** and **2** aligned in the nematic liquid crystal 5CB at 85 K following a 530 nm laser pulse (7 ns, 2 mJ). Spectra were measured with the liquid crystals oriented parallel (left column) and perpendicular (right column) to the magnetic field. Color scale: red = enhanced absorption, blue = emission, green = baseline.

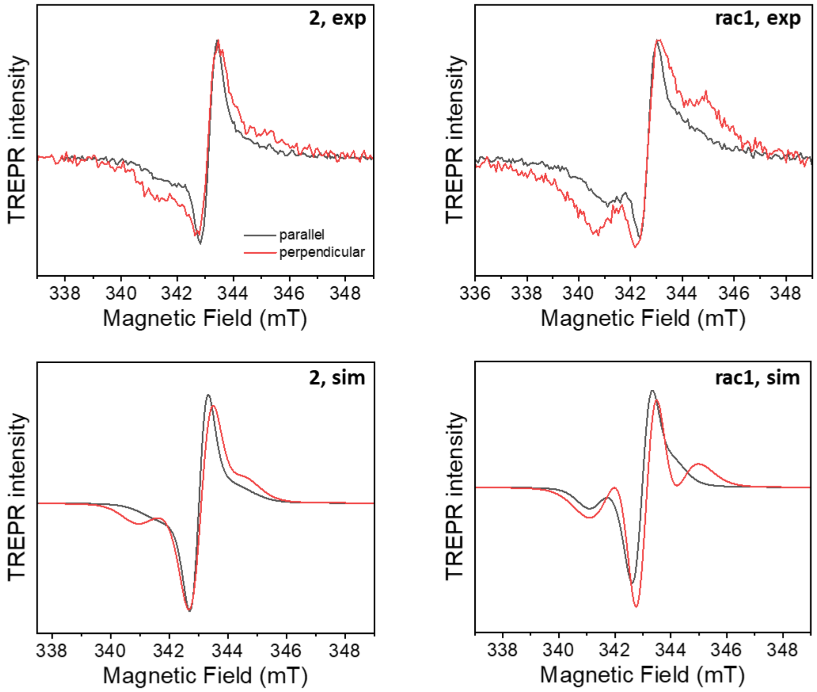

Figure S24. Comparison of experimental and simulated TREPR spectra of the **2** and **rac1** dyads aligned in the nematic liquid crystal 5CB, with orientations parallel (black lines) and perpendicular (red lines) to the external magnetic field. The spectra are normalized and horizontally shifted to facilitate comparison of spectral features.

Table S2. Computed $\mathbf{g_2}$ and $\mathbf{A_N}$ (MHz) values at the DFT level for dithia-aza[4]helicene+ and N-arylphenothiazine (see computational details in SI).

**Dithia-aza[4]helicene+**	[g_x g_y g_z]	g_{iso}
	[2.0020 2.0052 2.0066]	2.0046
	[A_{xx} A_{yy} A_{zz}](N)	A_{iso}(N)
	[6.426 6.603 41.115]	18.0478
**N-arylphenothiazine**	[g_x g_y g_z]	g_{iso}
	[2.0021 2.0057 2.0069]	2.0049
	[A_{xx} A_{yy} A_{zz}](N)	A_{iso}(N)
	[7.357 7.569 49.372]	21.433

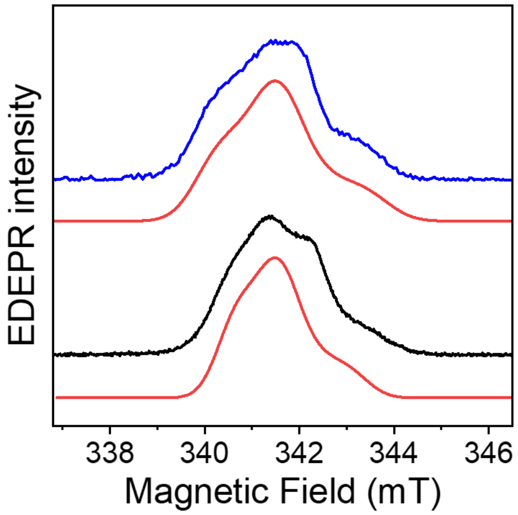

Figure S24. X-band echo-detected EPR spectra of the dithia-aza[4]helicene+ (blue line) and N-arylphenothiazine+ (black line) radical cations in CH_2Cl_2 at 85 K, along with spectral simulations (red lines) based on g-tensor and hyperfine tensor values obtained from DFT calculations.

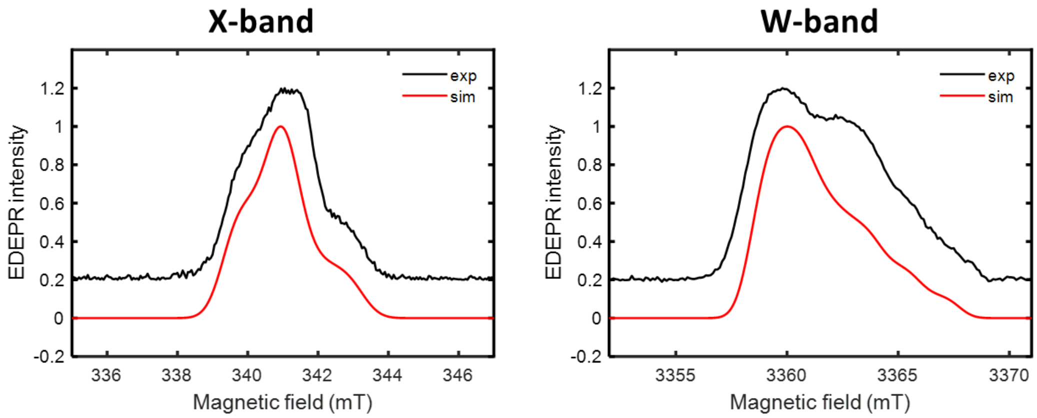

Figure S25. Comparison between the X-band and W-band echo-detected EPR spectra (black lines) of the dithia-aza[4]helicene^{+} radical cations in CH_2Cl_2 at 85 K, along with spectral simulations (red lines) based on g-tensor and hyperfine tensor values obtained from DFT calculations.

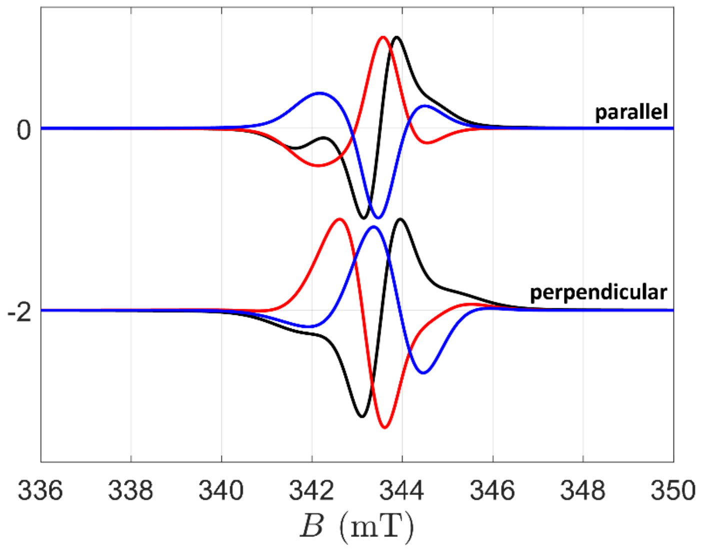

Figure S26. TREPR simulations performed using the same spin Hamiltonian parameters as in Figure 4, assuming absolute orientation of the dyads with respect to the external magnetic field. Following the nomenclature of Figure 4, parallel and perpendicular refer to the orientation of the dyad axis relative to the external magnetic field. The black trace corresponds to a singlet precursor without CISS, while the red and blue traces correspond to enantiomer 1 and enantiomer 2, respectively, assuming 100% CISS. The simulations show that, under absolute orientation, CISS effect is clearly observable for both parallel and perpendicular configurations. This arises because the chiral axis is not collinear with the charge-transfer direction.

7. References

(1) Cadena, D. M.; Sowa, J. K.; Cotton, D. E.; Wight, C. D.; Hoffman, C. L.; Wagner, H. R.; Boette, J. T.; Raulerson, E. K.; Iverson, B. L.; Rossky, P. J.; Roberts, S. T. Aggregation of Charge Acceptors on Nanocrystal Surfaces Alters Rates of Photoinduced Electron Transfer. *J. Am. Chem. Soc.* **2022**, *144* (49). https://doi.org/10.1021/jacs.2c09758.

(2) Rivero, D. S.; Pérez-Pérez, Y.; Perretti, M. D.; Santos, T.; Scoccia, J.; Tejedor, D.; Carrillo, R. Kinetic Control of Complexity in Multiple Dynamic Libraries. *Angewandte Chemie - International Edition* **2024**, *63* (29). https://doi.org/10.1002/anie.202406654.

(3) Greszler, S. N.; Reichard, H. A.; Micalizio, G. C. Asymmetric Synthesis of Dihydroindanes by Convergent Alkoxide-Directed Metallacycle-Mediated Bond Formation. *J. Am. Chem. Soc.* **2012**, *134* (5). https://doi.org/10.1021/ja2105043.

(4) Kim, K. H.; Seo, S. E.; Park, C. S.; Kim, S.; Lee, S.; Ryu, C. M.; Yong, D.; Park, Y. M.; Kwon, O. S. Open-Bandgap Graphene-Based Field-Effect Transistor Using Oligo(Phenylene-Ethynylene) Interfacial Chemistry. *Angewandte Chemie - International Edition* **2022**, *61* (41). https://doi.org/10.1002/anie.202209726.

(5) Pera, G.; Martín, S.; Ballesteros, L. M.; Hope, A. J.; Low, P. J.; Nichols, R. J.; Cea, P. Metal-Molecule-Metal Junctions in Langmuir-Blodgett Films Using a New Linker: Trimethylsilane. *Chemistry - A European Journal* **2010**, *16* (45). https://doi.org/10.1002/chem.201001181.

(6) Marqués-González, S.; Yufit, D. S.; Howard, J. A. K.; Martín, S.; Osorio, H. M.; García-Suárez, V. M.; Nichols, R. J.; Higgins, S. J.; Cea, P.; Low, P. J. Simplifying the Conductance Profiles of Molecular Junctions: The Use of the Trimethylsilylethynyl Moiety as a Molecule-Gold Contact. *Dalton Transactions* **2013**, *42* (2). https://doi.org/10.1039/c2dt31825c.

(7) Lupi, M.; Salmi, O.; Viglianisi, C.; Menichetti, S. A Lewis Base Hydrogen Bond Donor (LB/HBD) Organocatalytic Approach to Dithiabridged Triarylamine Hetero[4]Helicenes. *Adv. Synth. Catal.* **2023**, *365* (10), 1705–1712. https://doi.org/10.1002/adsc.202300238.

(8) Amorati, R.; Valgimigli, L.; Baschieri, A.; Guo, Y.; Mollica, F.; Menichetti, S.; Lupi, M.; Viglianisi, C. SET and HAT/PCET Acid-Mediated Oxidation Processes in Helical Shaped Fused Bis-Phenothiazines. *ChemPhysChem* **2021**, *22* (14), 1446–1454. https://doi.org/10.1002/cphc.202100387.

(9) Weller, A. Photoinduced Electron Transfer in Solution: Exciplex and Radical Ion Pair Formation Free Enthalpies and Their Solvent Dependence. *Zeitschrift fur Physikalische Chemie* **1982**, *133* (1). https://doi.org/10.1524/zpch.1982.133.1.093.

(10) Young, R. M.; Dyar, S. M.; Barnes, J. C.; Juríček, M.; Stoddart, J. F.; Co, D. T.; Wasielewski, M. R. Ultrafast Conformational Dynamics of Electron Transfer in ExBox 4+⊂Perylene. *Journal of Physical Chemistry A* **2013**, *117* (47), 12438–12448. https://doi.org/10.1021/jp409883a.

(11) Hartnett, P. E.; Margulies, E. A.; Matte, H. S. S. R.; Hersam, M. C.; Marks, T. J.; Wasielewski, M. R. Effects of Crystalline Perylenediimide Acceptor Morphology on Optoelectronic Properties and Device Performance. *Chemistry of Materials* **2016**, *28* (11), 3928–3936. https://doi.org/10.1021/acs.chemmater.6b01230.

(12) Coleman, A. F.; Chen, M.; Zhou, J.; Shin, J. Y.; Wu, Y.; Young, R. M.; Wasielewski, M. R. Reversible Symmetry-Breaking Charge Separation in a Series of Perylenediimide Cyclophanes. *Journal of Physical Chemistry C* **2020**, *124* (19), 10408–10419. https://doi.org/10.1021/acs.jpcc.0c02382.

(13) Kopp, S. M.; Nakamura, S.; Phelan, B. T.; Poh, Y. R.; Tyndall, S. B.; Brown, P. J.; Huang, Y.; Yuen-Zhou, J.; Krzyaniak, M. D.; Wasielewski, M. R. Luminescent Organic Triplet Diradicals as Optically Addressable Molecular Qubits. *J. Am. Chem. Soc.* **2024**, *146* (40), 27935–27945. https://doi.org/10.1021/jacs.4c11116.
